# Association between pain, neuropsychiatric symptoms, and physical function in dementia: a systematic review and meta-analysis

**DOI:** 10.1186/s12877-015-0048-6

**Published:** 2015-04-19

**Authors:** Annelore H van Dalen-Kok, Marjoleine JC Pieper, Margot WM de Waal, Albert Lukas, Bettina S Husebo, Wilco P Achterberg

**Affiliations:** 1 Department of Public Health and Primary Care, Leiden University Medical Centre, Hippocratespad 21 Post zone V0-P, PO Box 9600, Leiden, RC 2300 The Netherlands; 2 Department of General Practice & Elderly Care Medicine, VU University Medical Centre Amsterdam, van der Boechorststraat 7, Amsterdam, BT 1081 The Netherlands; 3 Malteser Hospital Bonn/Rhein-Sieg, Centre of Geriatric Medicine, Academic Hospital University of Bonn, Von-Hompesch-Straße 1, Bonn, 53123 Germany; 4 Department of Public Health and Primary Care, Centre for Elderly and Nursing Home Medicine, University of Bergen, Bergen, Norway; 5 Stavanger University Hospital, Bergen, Norway

**Keywords:** Pain, Dementia, Neuropsychiatric symptoms, Physical function, Associations

## Abstract

**Background:**

Pain, neuropsychiatric symptoms (NPS) and functional impairment are prevalent in patients with dementia and pain is hypothesized to be causal in both neuropsychiatric symptoms (NPS) and functional impairment. As the exact nature of the associations is unknown, this review examines the strength of associations between pain and NPS, and pain and physical function in patients with dementia. Special attention is paid to the description of measurement instruments and the methods used to detect pain, NPS and physical function.

**Methods:**

A systematic search was made in the databases of PubMed (Medline), Embase, Cochrane, Cinahl, PsychINFO, and Web of Science. Studies were included that described associations between pain and NPS and/or physical function in patients with moderate to severe dementia.

**Results:**

The search yielded 22 articles describing 18 studies, including two longitudinal studies. Most evidence was found for the association between pain and depression, followed by the association between pain and agitation/aggression. The longitudinal studies reported no direct effects between pain and NPS but some indirect effects, e.g. pain through depression. Although some association was established between pain and NPS, and pain and physical function, the strength of associations was relatively weak. Interestingly, only three studies used an observer rating scale for pain-related behaviour.

**Conclusions:**

Available evidence does not support strong associations between pain, NPS and physical function. This might be due to inadequate use or lack of rating scales to detect pain-related behaviour. These results show that the relationship between pain and NPS, as well as with physical function, is complicated and warrants additional longitudinal evaluation.

**Electronic supplementary material:**

The online version of this article (doi:10.1186/s12877-015-0048-6) contains supplementary material, which is available to authorized users.

## Background

Pain is common among older persons due to the increased prevalence of age-related diseases like osteoporosis and arthritis [1]. This also applies to patients with dementia living in nursing homes: around 50% is in pain [2,3].

Due to the changed perception of pain and loss of language skills in dementia, pain is often not communicated as such. In these patients, pain is often reported to be expressed as challenging behaviour (e.g. agitation or withdrawal) and is also known as neuropsychiatric symptoms (NPS) [4-6]. NPS includes depressive symptoms, agitated/aggressive behaviour, and psychotic symptoms like hallucinations and delusions [7].

NPS is highly prevalent: up to 80-85% of patients with dementia experience these symptoms [7-9] and they are one of the main reasons for institutionalisation [9,10]. The aetiology of NPS is multifactorial and includes neuropathological changes in the brain related to dementia and dementia severity, as well as unmet physical and psychological needs, physical illness (e.g. urinary tract infections), and pain [11].

Furthermore, pain influences the patient’s physical function, including sleep, nutrition, and mobility [12-15]. Therefore, physical inactivity and disability in patients with dementia may be an expression of pain, but can also be the cause of pain [16,17]. This illustrates that, due to its diverse presentation, the interpretation of potential signs and symptoms of pain in dementia is difficult; moreover, to date, most studies still report a systematic under-recognition and under-treatment of pain [18-20]. There is evidence for specific pain-related behaviour, such as increased wandering or irritability, but facial expressions, body movements, and vocalizations are also common [21]. These behaviours can help in the clinical decision-making process [22]. Consequently, in the last decades, measurement and assessment of pain in patients with dementia by means of observations of these behaviours have received increasing attention. However, clinicians still have insufficient tools to face the challenges in the diagnostics and treatment of pain in this vulnerable group, [22,23] and this may result in clinical indecisiveness. Nevertheless, there are validated measurement instruments available to detect pain in patients with dementia, such as the PACSLAC, DOLOPLUS-2, and the MOBID-2, based on observations [24,25]. Adequate use of these measurement instruments is of utmost importance in the management of pain.

Due to the challenges in the assessment and management of pain [26], people with dementia and NPS are more likely to receive antipsychotic drugs, despite the adverse side-effects like falls, somnolence and even death [27-29]. The latter underlines the importance of understanding the attributive effect of pain as a cause of NPS and decline in physical function. This would give healthcare workers more insight as to whether to target their treatment primarily on pain, NPS, disability, or on these conditions simultaneously.

Therefore, the aim of this systematic review is to assess the strength of associations between pain and NPS, and between pain and physical function, in patients with dementia. Special attention is paid to the description of measurement instruments and the method of detecting pain, NPS, and physical function to give clinical and scientific direction to the assessment and treatment of pain.

## Methods

### Study selection

This review was conducted following the PRISMA guidelines for systematic reviews [30]. A systematic search of the following databases was performed in March 2013: PubMed (Medline), Embase, Cochrane, Cinahl, PsychINFO, and Web of Science. In addition, the reference lists of the retrieved articles were screened. The following search terms (Additional file 1) were applied: Dementia AND Pain AND ((depression) OR (BPSD) OR (mobility) OR (sleep) OR (eating) OR (ADL)). Two reviewers, AvD and MP, independently, screened each title and abstract for suitability for inclusion; they decided independently on the eligibility of the article according to the predetermined selection criteria. Disagreement was resolved by consensus after review of the full article, or after the input of a third author (WA/MdW).

Articles that met the following criteria were included: patients with moderate to severe dementia (defined as a Mini Mental State Examination (MMSE) score of ≤18 or a Global Deterioration Scale (GDS) score of 5–7 [31]), description of data on pain, description of NPS, and/or physical function [eating, sleep, activities of daily living (ADL) and mobility]. For the purpose of this review, articles that described patients with mild to moderate dementia, but reported statistical data separately for the subgroup ‘moderate dementia’, were also included.

Eligible study designs included clinical trials, cohort, cross-sectional, observational, and longitudinal studies. Unless there was a clear description of the original data and baseline statistics, systematic reviews, qualitative studies, study protocols, (editorial) letters, case reports and randomised controlled trials (RCTs) were excluded. However, the reference lists of these articles were screened for eligible studies that were missed during the initial search. Only published data was included.

Excluded were articles that described patients who suffer from dementia resulting from Parkinson’s disease and Huntington’s disease, AIDS dementia complex, and Creutzfeldt-Jakob Syndrome. Furthermore, we excluded articles that did not report correlation coefficients or odds ratio’s (OR), or when the articles did not provide sufficient information to calculate the OR ourselves. No time range or language restrictions were used.

### Data extraction

Data were independently extracted by two reviewers (AvD and MP). A data extraction form was designed before extracting data from the included articles.

We recorded data on: study characteristics (design, country, setting, study population), pain and NPS measurement, prevalence of pain, and correlations of pain, NPS, and physical function. Where possible we present unadjusted associations, as these reflect the presence of co-occurrence as perceived by the caregivers. In addition we calculated the OR ourselves if not reported. These ORs are reported as self-calculated odds ratio (SOR).

Furthermore, we recorded data on the use of rating scales to measure pain, NPS and physical function, as well as the method of detection. For example, if pain was measured with a rating scale for observational behaviours indicating pain and who performed the observation, i.e. a research nurse, a professional or patient’s proxy.

### Quality assessment

The methodological quality assessment of the included cross-sectional and longitudinal studies was based on previously developed checklists [32,33]. Two reviewers (AvD and MP) independently assessed the quality of each study. Disagreement was resolved by consensus or after input of a third author (MdW/WA). The maximum total score possible for cross-sectional studies was 12 points and for longitudinal studies 14 points. Cross-sectional studies that scored 0–4 points were considered to be of ‘low quality’, scores of 5–9 to be of ‘moderate quality’, and scores of ≥10 points were considered to be of ‘high quality’. For longitudinal studies, scores of 0–5 points were considered to be of ‘low quality’, scores of 6–11 points to be of ‘moderate quality’, and scores of ≥12 points were considered to be of ‘high quality’. See Additional file 2 for a more detailed overview of the awarded points and scores to the articles.

### Scoring items

We selected items relevant for the assessment of observational studies, such as a description of a clearly stated objective, use of valid selection criteria, a response rate of ≥80%, valid/reproducible measurement of the outcome, adjusting for possible confounders, and the presentation of an association. One point was awarded for each questions answered with ‘yes’ and 0 points for every ‘no’ or ‘?’. We added two questions concerning the study objective and population: i) was the selected objective similar to our objective, and ii) was the study population a selected population.

Furthermore, we wanted the quality assessment to reflect the ability to study our research objective. Therefore, we added a few items focusing on the measurement of pain, i.e. the use of specific rating scales, the method of detection, and information about the rater. Awarded points ranged from 0–2.

Additionally, two questions were added to the quality assessment for the longitudinal studies: i) was there major and selective loss to follow-up, and ii) was there a sufficiently long follow-up period [32,33]. Again, 1 point was awarded for each questions answered with ‘yes’ and 0 points for each ‘no’ or ‘?’.

### Statistical analysis

To provide a more comprehensive overview of the association between pain, NPS and physical function, the available ORs are displayed in forest plots (using the program Review Manager 5.2) including the pooled ORs using a random effects model.

## Results

### Selected articles

**Figure 1:**
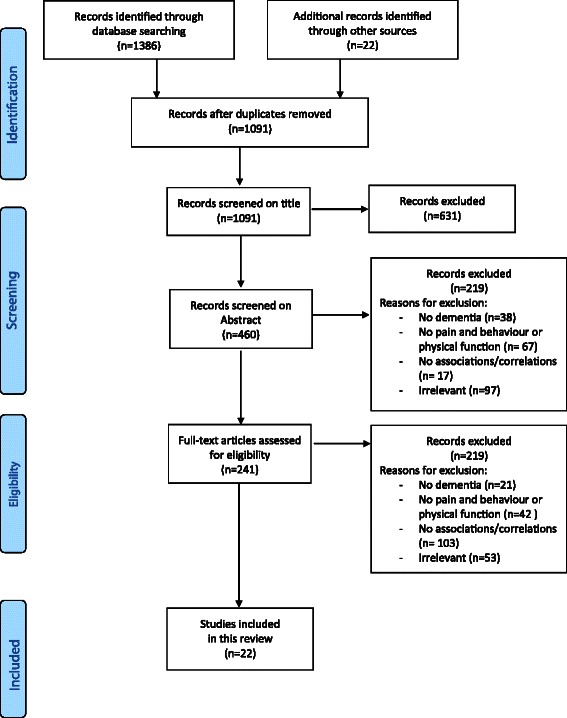
PRISMA flowdiagram of the inclusion of studies.

The literature search yielded 1386 articles; 786 from PubMed (Medline), 304 from Embase, 77 from Cinahl, 57 from PsychINFO, 96 from Cochrane, and 66 from Web of Science. Additionally, 22 articles were retrieved from other sources (mainly through checking the reference lists). After removing duplicates, 1091 unique articles were identified. After carefully screening the titles, abstracts and full text, 22 publications met the inclusion criteria and were included in the present review (Figure 1).

### Description of included studies

All included articles were published between 2002 and 2013.

Of these 22 articles, eight articles illustrate correlates of pain with specified behavioural problems such as delusions/psychosis [3,34], anxiety [35], wandering [3,36], and resistance to care [3,37,38]. Furthermore, seven articles described associations between pain and unspecified behavioural problems, such as behavioural/psychiatric problems and dysfunctional behaviours [3,4,39-43]. It was not clarified which types of NPS were embedded in this term.

Eleven articles described the association between pain and depression [4,8,34,35,43-49] and eight articles between pain and aggression/agitation [8,34,36,38,47,48,50,51] In addition, relationships between pain and physical function (e.g. ADL dependency and mobility) were described in ten articles [3,4,39,40,43,44,46,48,49,52]. The characteristics of these articles are presented in Table 1.

**Table 1:** Characteristics of the included studies

First author	Country, setting	Dementia	Population: selection on pain, NPS or function?	Quality of study**
**Ahn 2013** **[** 36 **]**	USA, nh	Moderate dementia, mean MDS cognitive performance scale 3.17 (SD 1.52)	Age ≥ 65 years, excluded when comatose	10
**Bartels 2003** **[** 8 **]**	USA, ltc	Dementia, AD or signs of chronic stable cognitive impairment (in chart or MDS)	At risk for (or having) pressure ulcers	4
**Black 2006** **[**39]	USA, nh	Advanced dementia, SIRS mean 10.3 (SD 6.7), AD 58%	Palliative care (life expectancy ≤6 months)	6.5
**Brummel-Smith 2002** **[** 40 **]**	USA, nh	Moderate to severe dementia, MMSE mean 16.8 (SD 5.6) for 92 subjects	Age ≥ 55 years, had to have pain assessment, able to self-report on their level of pain	7
**Cipher 2004** **[** 4 **]**	USA, ltc	Moderate dementia, mean NCSE 0.10 (SD 0.91)	Referral to clinical psychologist due to change in cognitive functioning, emotional distress, or behavioural dysfunction associated with dementia	7.5
**Cipher 2006** **[** 41 **]**	USA, ltc	Dementia, mild 40%, moderate 41% and severe 19%, according to FAST (Reisberg) NCSE	Referral to clinical psychologist due to change in cognitive functioning, emotional distress, or behavioural dysfunction associated with dementia	7.5
**D’Astolfo 2006** **[** 44 **]**	Canada, ltc	In 4% no dementia with MMSE>25, mild dementia 27%, moderate 44%, severe 25%	Admission in ltc at least 6 months to allow for patient charts to be completed	7
**Gruber-Baldini 2005** **[** 45 **]**	USA, nh and residential care/assisted living	Dementia, mild 14%, moderate 26% and severe 61%, according to MMSE or MDS-COGS.	Random sample aged ≥ 65 years (complete response 60%)	8.5
**Kunik 2005** **[** 34 **]**	USA, va outpatients	Dementia, mild 46%, moderate 39%, severe 11%, according to DRS.	Veteran outpatients, not in LTC-facilities, with available caregiver	8.5
**Leonard 2006** **[** 50 **]**	USA, nh	Dementia according to CPS-MDS dataset	At least one comprehensive MDS assessment, age ≥ 60 years	9
**Leong 2007** **[** 35 **]**	Singapore, nh	Dementia with 33% mild (MIC) and 41% severe (SIC) cognitive impairment, according to AMT	No recent change in cognitive status, age ≥ 65 years. Here report of *communicative* subgroup *with dementia* (thus excluding 53 and including 125 of 358).	8.5
**Lin 2011** **[** 46 **]**	Taiwan, nh	Dementia, 39% profound or end-stage dementia, according to CDR-C.	Admission at least 1 month	12
**Morgan 2012** **[** 47 **]**	USA, Veterans Administration Medical Centre, longitudinal study	Dementia, DemRS2 mean 4.12 (SD 2.79)	> 60 years, no aggressive behaviour in past year, no residence in nh and caregiver > 8 hrs a week, no onset of aggression before first follow-up (at 5 mo)	9.5
**Norton 2010** **[** 42 **]**	USA, nh	Dementia, MMSE mean 6.4 (SD 6.7)	Verbal disruption (BEHAVE-AD >= 1.5), age ≥ 55 years, passed audiological assessment, and life expectancy >6 mo	9
**Shega 2005** **[** 48 **]**	USA, outpatient geriatrics clinic	Dementia, MMSE mean 16.6 (SD 7.2)	Patient-caregiver dyad with pain-report on same day (77% of original sample)	9.5
**Shega 2010** **[** 49 **]**	Canada, community dwelling	Cognitive impairment, 3 MS, mild to moderate dementia 18.5%	Community dwelling people aged ≥ 65 years, within one inclusion wave a pain self-assessment was incorporated	9
**Torvik 2010** **[** 52 **]**	Norway, nh	No (13%), mild (46%) or moderate (41%) cognitive impairment, according to MMSE.	MMSE > 11, aged ≥ 65 years (inclusion and response 35% of total sample). Communicative patients	6.5
**Tosato 2012** **[** 3 **]**	EU and Israel, nh	Cognitive impairment, mild-moderate 55% and severe 45%, according to CPS	Several countries	11.5
**Volicer 2009** **[** 37 **]**	Netherlands, nh/residential home	Dementia, according to MDS-CPS	Dependent in decision making, aged ≥ 65 years	11
**Volicer 2011** **[** 51 **]**	Netherlands, nh, longitudinal study	Dementia, according to MDS	Availability of 4 quarterly MDS assessments within period of 15 months, aged ≥ 65 years	12
**Williams 2005** **[** 43 **]**	USA, nh and residential care/assisted living	Dementia, with 29% MMSE>10 and MDS-COGS >2-4	Available pain data, aged ≥ 65 years	10
**Zieber 2005** [38]	Canada, ltc	Moderate to severe cognitive impairment, according to FAST (Reisberg) score 6-7	Residents with continuous nursing care because of significant physical and/or cognitive impairments (‘nh-level’)	8

**Abbreviations:** nh, nursing home; MDS, Minimum Dataset; ltc, long term care facility; AD, Alzheimer’s Disease; SIRS, The Severe Impairment Rating Scale; MMSE, Mini Mental State Examination; NCSE, Neurobehavioural Cognitive Status Examination; FAST, Functional Assessment Staging; MDS-COGS, Minimum Dataset Cognition Scale; va, veterans affairs; DRS, Dementia Rating Scale; CPS, Cognitive Performance Scale; AMT, Abbreviated Mental Test; CDR-C, Clinical Dementia Rating Scale-Chinese Version; Dem-RS2, Dementia Rating Scale 2; SD, Standard Deviation; BEHAVE-AD, Behavioural Pathology in Alzheimer’s disease.

**Based on checklists from van der Windt et al. [52,53] Higher scores indicate higher quality (range observational studies 0–12, range longitudinal studies 0–14) Observational studies that scored ≥10 point were considered ‘high quality’. Longitudinal studies that scored ≥12 points were considered ‘high quality’.

Most of the studies described patients aged ≥ 65 years, who were mainly diagnosed with moderate to severe dementia and resided in long-term care facilities throughout the USA [4,8,34,36,39-43,45,47,48,50]. Three studies took place in Europe [3,51-53], three studies in Canada [38,44,49], and two studies took place in Asia [35,46].

Of the 20 cross-sectional studies, five studies were considered to be of high quality [3,36,37,43,46]. The remaining 15 studies were of low to moderate quality. Of the two longitudinal studies, that of Volicer et al. was considered to be of high quality [51] (Table 1).

Five studies described the use of selection criteria, mostly on NPS, and in eight other studies there might have been an indirect (unintentional) selection on pain, NPS or functioning. For instance, an indirect selection on pain by including patients with pressure ulcers [8].

Eight articles described the same study populations, sometimes with additional selection criteria, e. g. the two articles by Cipher et al. [4,41]. Kunik et al. and Morgan et al. used data from a large longitudinal study on the causes and consequences of aggression in persons with dementia. Another two articles extracted data from the Dementia Care project of the Collaborative Studies of Long-Term Care [43,45] and two articles derived their data from the same Minimum Dataset 2.0 for nursing home care [37,51].

### Overview of measurement instruments

Table 2 describes how pain, NPS, and physical function were measured.

**Table 2:** Measurements of pain, neuropsychiatric symptoms and physical function

	Measurement of pain		Measurement of neuropsychiatric symptoms		Measurement of function	
First author	Rating scale	Method of detection	Rating scale	Method of detection	Rating scale	Method of detection
**Ahn 2013** **[** 36 **]**	MDS pain severity scale, combining pain frequency and pain intensity	Self-report, if not possible staff report based on proxy reports	MDS subscales; wandering-item, aggression behaviour scale (ABS), challenging behaviour profile (CBP) agitation subscale	Patient self-report, proxy and professional	MDS-ADL long form (7 items)	Staff observation
**Bartels 2003** **[** 8 **]**	No use of rating scale	Data collection instrument (3-month period), raters unknown	MDS for depression	Medical records	MDS (number of ADLs)	Medical records
**Black 2006** **[** 39 **]**	GMPI pain and suffering subscale	Part of neuropsychological evaluation by a licensed clinical geropsychologist	-GDS-15 “-26 dysfunctional behaviours with scores “1-7”	Part of neuropsychological evaluation by a licensed clinical geropsychologist	PRADLI	Part of neuropsychological evaluation by a licensed clinical geropsychologist
**Cipher 2006** **[** 41 **]**	GMPI	Part of neuropsychological evaluation by a licensed clinical geropsychologist and each instrument was administered after interviewing the resident, nursing staff and family members	GLDS, 19 categories with scores 1-7	Part of neuropsychological evaluation by a licensed clinical geropsychologist and each instrument was administered after interviewing the resident, nursing staff and family members Medical records, preceding 6 to max 26 Months	GLDS	Part of neuropsychological evaluation by a licensed clinical geropsychologist and each instrument was administered after interviewing the resident, nursing staff and family members
**D’Astolfo 2006** **[** 44 **]**	No use of rating scale	Medical records, preceding 6 to max 26 months	No use of rating scales		No use of rating scale	Medical records Ambulatory status: independent, requires assistance, wheel chair (or bedridden n=1)
**Gruber-Baldini 2005** **[** 45 **]**	PGC-PIS, score ≥ 2	Rating by supervisory staff member	CSDD CMAI	Rating by supervisory staff member	MDS; activities of daily living scale, SMOI	Rating/observation by supervisory staff member
**Kunik 2005** **[** 30 **]**	PGC-PIS, item on level of pain in previous week, scores 1-6	Interview with patient and proxy by trained interviewer/research assistant	CMAI HAM-D NPI (subdomains delusion/hallucinations)	Interview with patient and proxy by trained interviewer/research assistant	-	-
**Leonard 2006** [50]	MDS pain burden using a 4-level composite score based on pain frequency and intensity	-	MDS (Physical aggression: MDS item ‘others were hit, shoved, scratched, sexually abused’; Depression: MDS score≥3 on sum of 9 items, e.g. ‘being sad’, ‘making negative statements’, ‘persistent anger with self or others’, ‘pained facial expressions’. (At least once in week before))	-	-	-
**Leong 2007** **[** 35 **]**	PAINAD for non-communicative patients	Interviews with patient and staff member by professionals for communicative patients	Depression with GDS-15 or STAI Anxiety with Cornell	Self-report or staff report	AAS	Not reported
**Lin 2011** **[** 46 **]**	PAINAD-Chinese version	Observation immediately following instances of routine care by principal investigator and research assistant	No use of rating scales	Medical records and observations by professional	No use of rating scale	Medical records and observation by professional
**Morgan 2012** **[** 47 **]**	PGC-PIS worst pain item	Not reported	CMAI aggression subscale CMAI non-aggressive physical agitation subscale HAM-D depression	Not reported	-	-
**Norton 2010** **[** 42 **]**	PPQ, intensity item, 10–14 day baseline	Primary CNA and data used from medical records	RMBPC-NH, selection of 3 need driven behaviours, BEHAVE-AD	Primary CNA and unit staff	PSMS	Nurses and trained research assistants
**Shega 2005** **[** 48 **]**	VDS, 1 item on presence and severity of pain ‘right now’	Interviews with patients and caregivers by trained research assistant	GDS-15 CMAI	Interview patient and proxy	KATZ IADL	Interview patient and proxy
**Shega 2010** **[** 49 **]**	VDS, 5 point, ‘pain past 4 weeks’	Interviews with patient by trained research assistant	Mental Health screening questionnaire; 5-item and 6 point scale	Interview with patient by trained research assistant	OARS/IADL; 3 point scale	Interview patient by trained research assistant
**Torvik 2010** **[** 48 **]**	VRS, 4 point, ‘pain right now’	Patient self-report	DQoL, 29-items on 5 domains: self-esteem, aesthetics, positive affect, negative affect, belonging	Not reported	Barthel	Self-report and medical records
**Tosato 2012** **[** 3 **]**	InterRAI LTCF	InterRAI LTCF questions and observation of behaviour, any type of pain or discomfort of the body in previous 3 days by trained (research) staff	InterRAI LTCF 5 behavioural symptoms, previous 3 days	Not reported	MDS ADL Hierarchy Scale	Data recorded by study physicians
**Volicer 2009** **[** 37 **]**	MDS-RAI pain frequency (item J2a)	Combination of physical examination, patient history, observation, consultation caregiver and medical records by staff	MDS Depression Rating Scale MDS item J1e for delusions MDS item J1i for hallucinations	Combination of physical examination, patient history, observation, consultation caregiver and medical records by staff	-	-
**Volicer 2011** **[** 51 **]**	MDS	Combination of physical examination, patient history, observation, consultation caregiver and medical records by staff	MDS items I1ee, E1a, E1d, E1f, E1b, E1i, E1l, E1m for depression MDS for delusions and hallucinations MDS items B5b, E1b, E4aa, E4da for agitation	Combination of physical examination, patient history, observation, consultation caregiver and medical records by staff	-	-
**Williams 2005** **[** 43 **]**	PGC-PIS, score ≥2, and 0–10 pain numeric rating scale	Registered nurses or licensed practical nurses and interview with overseeing supervisor	CSDD, score ≥7 CMAI, any behaviour at least weekly	Rating by care supervisors, registered nurses and licensed practical nurses	MDS-ADL, APAS SMOI	Rating by care supervisors, registered nurses and licensed practical nurses
**Zieber 2005** **[** 38 **]**	DS-DAT, and a 7-point pain rating scale	Trained facility nurses, palliative care nurse consultants	PAS	Trained facility nurses	-	-

**Abbreviation:** MDS, Minimum Dataset; ADL, Activities of Daily Living; GMPI, Geriatric Multidimensional Pain and Illness Inventory; GDS-15, Geriatric Depression Scale-15 short version; PRADLI, Psychosocial Resistance to Activities of Daily Living Index; GLDS, Geriatric Level of Dysfunction Scale; PGC-PIS, Philadelphia Geriatric Centre Pain Intensity Scale; CSDD, Cornell Scale for Depression in Dementia; CMAI, Cohen-Mansfield Agitation Inventory; SMOI, Structured Meal Observational Instrument; HAM-D, Hamilton Rating Scale for Depression; NPI, Neuropsychiatric Inventory; PAINAD, Pain Assessment in Advanced Dementia; STAI, State-Trait Anxiety Inventory; AAS, Adjusted Activity Scale; PPQ, Proxy Pain Questionnaire; CNA, Certified Nursing Assistant; RMBPC-NH, Revised Memory and Behaviour Problems Checklist-Nursing Home; BEHAVE-AD, Behavioural Pathology in Alzheimer’s disease; PSMS, Physical Self Maintenance Scale; VDS, Verbal Descriptor Scale; KATZ, Index of Independence in Activities of Daily Living; IADL, Instrumental Activities of Daily Living; OARS/IADL, Older Americans Recourses and Services/Instrumental Activities of Daily Living; VRS, Verbal Rating Scale; DQol, Dementia Quality of life; APAS, Albert Patient activity Scale; DS-DAT, Discomfort Scale - Dementia of Alzheimer Type; PAS, Pittsburgh Agitation Scale.

### Measurement of pain

Three articles describe rating scales for observational behaviours indicating pain; both scales are validated for patients with moderate to severe dementia, i.e. the PAINAD [35,46] and DS-DAT [38]. The remaining articles describe other methods to measure pain (Additional file 3); some articles used the MDS dataset [3,36,37,50,51] and others used a variety of rating scales, e.g. the Faces Pain Scale [40], the Geriatric Multidimensional Pain and Illness Inventory [4,41], the Proxy Pain Questionnaire [52] and the Philadelphia Geriatric Center Pain Intensity Scale [34,43,45,47]. The Verbal Descriptive Scale and Verbal Rating Scale were also used to measure pain, sometimes combined with self-report [48,49,52]. Three articles used no rating scales to measure pain; they extracted data form patient’s medical records [8,44] and interviewed patient’s proxy and/or healthcare worker [39].

Additional file 3 provides a complete overview of the methods used.

### Measurement of NPS

There was no uniform way of reporting NPS. The terms ‘behavioural symptoms’, ‘psychiatric symptoms’, and ‘disruptive behaviour’ were commonly used to describe any type of behavioural symptoms, e.g. agitation, depression, and anxiety [3,4,39-41].

The most common type of reported NPS was depression, followed by symptoms such as wandering, resistance to care, and verbal or physical abuse [36,37,42]. Four articles used no rating scales to measure NPS; they screened medical records instead [8,39,44,46]. Nine articles used more than one rating scale simultaneously to asses NPS [4,34,35,42,43,45,47,49,50]. Eight of those articles used rating scales to assess behaviour in patients with dementia; the Cornell Scale for Depression in Dementia [43,45], the Cohen-Mansfield Agitation Inventory [34,43,45,47,49], Behavioural Pathology in Alzheimer’s disease [42], and the Neuropsychiatric Inventory [34] (Table 2). One article used the Mental Health screening questionnaire to assess depressed mood [49]. The MDS Dataset was also frequently used [8,36,37,50,51].

### Measurement of Physical Function

Physical function was described in eleven articles [3,4,39,40,43-46,48,49,52].

Types of physical function that were reported in the articles are malnourishment [39,43,45], ADL dependency [3,4,40,43,49,52], and mobility [43,44,46].

Five articles used the MDS-ADL scale for measuring patient’s physical function (Table 2). This was also the most frequently used measurement [3,8,36,43-45].

### Associations between pain, NPS and physical function

Tables 3, 4, 5 and 6 describe the associations between pain, NPS, and physical function.

**Table 3:** Correlates of pain with depression

First author	N	Pain: prevalence	Depression: prevalence	Correlates of pain with depression	Quality of study
**Bartels 2003** **[** 8 **]**	1836	Pain 27%	Depression 32%	**SOR 1.6 (95% CI:** **1.3-2.0)**	4
**Cipher 2004** **[** 4 **]**	234	Persistent pain 72%	Depression (GDS-15) mean 7.8 (SD 3.12)	Correlations with GMPI ‘pain and suffering’ **r=0.13 (p<0.05)** with GDS-15 depression	7.5
**D’Astolfo 2006** **[** 44 **]**	140	Pain 64% (musculoskeletal pain 40%)	Depression 16%	SOR 1.3 (95% CI: 0.5-3.5) (analyses in sample of no dementia-severe dementia)	7
**Gruber-Baldini 2005** **[** 45 **]**	328	High pain 21%	Depression 23%	**SOR 3.1 (95% CI: 1.7-5.5)** (in n=328)	8.5
**Kunik 2005** **[** 34 **]**	99	Pain mean (PGC-PIS) 2.4 (SD 1.2)	Depression (HAM-D) mean 7.7 (SD 6.1)	**r=0.49 (p ≤0.01)**	8.5
**Leong 2007** **[** 35 **]**	225	Pain 44%; chronic pain 34%	Depression 61%	**SOR 3.2 (95% CI: 1.8-5.9)**	8.5
**Lin 2011** **[** 46 **]**	112	Observed pain 37% (PAINAD >= 2)	Depression 5%	OR=1.2 (95% CI: 0.19-7.26)	12
**Morgan 2012** **[** 47 **]**	171	Worst pain mean 1.91 (SD 1.53)	Depression (HAM-D) mean 6.16 (SD 5.28)	Baseline: r = 0.30 (n.s.)	9.5
**Shega 2005** **[** 48 **]**	115	Any current pain self-report 32%, caregiver report 53%	Depression (GDS-15) mean 3.1 (SD 2.7)	*For self-report pain* SOR 1.5 (95% CI: 0.6-3.7) *For caregiver pain* report: SOR 0.5 (95% CI: 0.2-1.1) with patient depression	9.5
**Shega 2010** **[** 49 **]**	5549	Moderate or greater pain: 35.8%	Depressed mood 37.3%	**OR=1.69 (95% CI: 1.18-2.44)** with depressed mood (Adjusted for demographics)	9
**Williams 2005** **[** 43 **]**	331	Pain 21%, in nh 23%, in rc/al 20% *(self-report for subgroup mmse>10 was: 39% and 25%)*	Depressed 23%	**OR=2.3 (1.1-4.8)** and **AOR=2.9 (1.2-7.2)** (Adjusted for: sex, race, age, cognitive status, number of 10 comorbidities, impairments of 7 activities of daily living)	10

**Abbreviations:** SOR, Self-Calculated Odds Ratio; SD, Standard Deviation; r, correlation coefficient; AOR, Adjusted Odds Ratio; OR, Odds Ratio; n.s., not significant; GMPI, Geriatric Multidimensional Pain and Illness Inventory; PGC-PIS, Philadelphia Geriatric Centre Pain Intensity Scale.

**Table 4:** Correlates of pain with agitation/aggression

First author	N	Pain: prevalence	Agitation/aggression: prevalence	Correlates of pain with agitation/aggression	Quality of study
**Ahn 2013** **[** 36 **]**	56577	Not reported	Aggression 24% Agitation 24%	**AOR 1.04 (95% CI: 1.01-1.08)** with aggression **AOR 1.17 (95% CI: 1.13-1.20)** with agitation *Subsample without use of psychotropic medication* **AOR 1.07 (95% CI: 1.01-1.15)** with aggression **AOR 1.16 (95% CI: 1.08-1.25)** with agitation (Adjusted for cognition, ADL, sociodemographics)	10
**Bartels 2003** **[** 8 **]**	1836	Pain 27%	Agitation 44%,	SOR 1.1 (95% CI: 0.9-1.4) with agitation	4
**Kunik 2005** **[** 34 **]**	99	Pain mean 2.4 (SD 1.2)	Agitation (CMAI) mean 14.3 (SD 4.1)	**r=0.20 (p≤0.05)** with aggression	8.5
**Leonard 2006** **[** 50 **]**	103344	Pain 24%; mild pain 15%, moderate to severe pain 9%	Physical aggression 7%	**SOR 0.8 (95% CI: 0.8-0.9)** for pain burden and physical aggression	9
**Morgan 2012** **[** 47 **]**	171	Worst pain mean 1.91 (SD 1.53)	Non aggressive physical agitation (CMAI) mean 12.14 (SD 4.50)	Baseline: r = 0.06 (n.s.) with aggression Follow-up: depression indirectly predicted onset of aggression, through pain	9.5
**Shega 2005** **[** 48 **]**	115	Any current pain self-report 32%, caregiver report 53%	Agitation (CMAI) mean 46.9 (SD 18.9),	*For self-report pain* no association with agitation (p>0.05) *For caregiver pain report* p=0.04 with agitation	9.5
**Volicer 2011** **[** 51 **]**	1101	Any pain 49%	Agitation (score>0, range 0–5) 76%	**r=0.22 to 0.26 (p<0.001)** with agitation (Range of correlations scores over 4 periods.) Follow-up: Longitudinal changes in agitation scores are related to changes in depression score but not to pain.	12
**Zieber 2005** **[** 38 **]**	58	Not reported	Not reported	**r=0.51 (p<0.01)** for DS-DAT scores and agitation (PAS-total) *Pain rating by palliative care nurse consultants*: **r=0.49 (p<0.01)** with agitation (PAS-total) *Pain rating by facility nurse*: **r=0.28 (p<0.05)** with agitation (PAS-total)	8

**Abbreviations:** AOR, Adjusted Odds Ratio; ADL, Activities of Daily Living; SOR, Self-Calculated Odds Ratio; SD, Standard Deviation; r, correlation coefficient; n.s, not significant; CMAI, Cohen Mansfield Agitation Inventory; DS-DAT, Discomfort Scale - Dementia of Alzheimer Type; PAS, Pittsburgh Agitation Scale.

**Table 5:** Correlates of pain with neuropsychiatric symptoms

Correlates of pain and Specified NPS					
*First author*	*N*	*Pain: prevalence*	*Neuropsychiatric symptoms: prevalence*	*Correlates of pain with NPS*	*Quality of study*
**Ahn 2013** **[** 36 **]**	56577	Not reported	Wandering 9%	**AOR 0.77 (95% CI: 0.73-0.81)** with wandering *Subsample without psychotropic medication* **AOR 0.72 (95% CI: 0.63-0.83)** with wandering (Adjusted for cognition, ADL, sociodemographics)	10
**Kunik 2005** **[** 34 **]**	99	Pain mean 2.4 (SD 1.2)	Delusions/hallucinations mean 0.35 (SD 0.48)	**r=0.15 (p>0.05)** with psychosis	8.5
**Leong 2007** **[** 35 **]**	225	Pain 44%, chronic pain 34%	Anxiety 48%	**SOR 1.8 (95% CI: 1.0-3.0)** with anxiety	8.5
**Norton 2010** **[** 42 **]**	161	Not reported	BEHAVE-AD mean 6..4 (SD 29.2) RMBPC-NH mean 1.45 (SD 0.64)	r=0.15 (p=0.08) for pain intensity and emotional behaviour problems r=0.05 (p=0.58) for pain intensity and resistiveness to care	9
**Torvik 2010** **[** 52 **]**	106	Current pain in total group 55%, in cognitive impaired group 52%	Negative affect index (DQoL) mean 2.0 (SD 0.75), positive affect/humour index (DQoL) mean 3.4 (SD 0.9)	**p<0.01** for current pain and negative affect p=0.11 for current pain and with positive affect/humour	6.5
**Tosato 2012** **[** 3 **]**	2822	Any pain 19% (moderate/severe/excruciating pain 13%)	Behavioural symptoms 37% Psychiatric symptoms 21%	**AOR=0.74 (95% CI: 0.55-1.0)** with wandering **AOR=1.4 (95% CI: 1.08-1.8)** with resistance to care **AOR 1.5 (95% CI: 1.07-2.03)** with delusions AOR 1.06 (95% CI: 0.80-1.41) with verbal abuse AOR 1.08 (95% CI: 0.75-1.55) with physical abuse (Adjusted for age, gender, country, cognitive impairment, number of diseases, ischemic heart disease, stroke, falls, communication problems, and a flare-up of a chronic or recurrent condition)	11.5
**Volicer 2009** **[** 37 **]**	929	Daily pain 29%, less than daily pain 19%	Verbally abusive not easily altered 2%, physically abusive not easily altered 12%, Delusions 8%, Hallucinations 9%	r=0.07 (p=0.03) for pain frequency and verbal abuse AOR=0.9(p=0.53) with resisting care AOR=0.7 (p=1.2) with verbal abuse AOR=0.7 (p=0.16) with physical abuse (Both multivariate models among others controlled for resisting care.)	11
**Zieber 2005** **[** 38 **]**	58	Not reported	Not reported	**r=0.46 (p<0.01)** for DS-DAT scores and resisting care **r=0.42 (p<0.01)** for DS-DAT scores and aberrant vocalization *Pain rating by palliative care nurse consultants*: **r=0.51 (p<0.01)** with resisting care **r=0.40 (p<0.01)** with aberrant vocalizations *Pain rating by facility nurse*: **r=0.48 (p<0.01)** with resisting care **r=0.065 (p<0.63)** with aberrant vocalizations	8
**Correlates of pain and Unspecified NPS**					
**Black 2006** **[** 39 **]**	123	Pain 63%	Psychiatric disorders or behaviour problems 85%, behaviour problems 67%	SOR 1.9 (95% CI: 0.7-5.3) with psychiatric/ behaviour problems SOR 1.2 (95% CI: 0.5-2.5) with behaviour problems	6.5
**Brummel-Smith 2002** **[** 40 **]**	104 (excluding those unable to self-report pain)	Moderate-severe pain 60% No-mild pain 40% 50 subject unable to answer	≥1 disruptive behaviours (wandering, verbal disruption, physical aggression, regressive behaviour, hallucinations) 70% in dementia sample n=154	SOR 1.8 (95% CI: 0.8-4.0) with ≥1 disruptive behaviour	7
**Cipher 2004** **[** 4 **]**	234	Persistent pain 72%	Dysfunctional behaviours mean 4.4 (SD 0.76)	**r=0.22 (p<0.05)** with dysfunctional behaviours	7.5
**Cipher 2006** **[** 41 **]**	277	Acute pain 29% Chronic pain 59%	-	**r=0.18 (p<0.05)** with GLDS mean behavioural intensity	7.5
**Norton 2010** **[** 42 **]**	161	Not reported	BEHAVE-AD mean 61.4 (SD 29.2) RMBPC-NH mean 1.45 (SD 0.64)	r=0.18 (p=0.03) for pain intensity and disruptive behaviour problems	9
				r=0.05 (p=0.53) for pain intensity and global need driven behaviours	
**2012** **[** 3 **]**	2822	Any pain 19% (moderate/severe/excruciating pain 13%)	Behavioural symptoms 37% Psychiatric symptoms 21%	**AOR=1.4 (95% CI: 1.04-1.8)** with socially inappropriate behaviour (Adjusted for age, gender, country, cognitive impairment, number of diseases, ischemic heart disease, stroke, falls, communication problems, and a flare-up of a chronic or recurrent condition)	11.5
**Williams 2005** **[** 39 **]**	331	Pain 21%, in nh 23%, in rc/al 20% *(self-report for subgroup mmse>10 was higher: 39% and 25%)*	Behavioural symptoms 58%	OR=1.1 (95% CI: 0.49-2.29) and AOR=1.2 (95% CI: 0.57-2.36) with behavioural symptoms	10
				(Adjusted for: sex, race, age, cognitive status, number of 10 comorbidities, impairments of 7 activities of daily living)	

**Abbreviations:** AOR, Adjusted Odds Ratio; ADL, Activities of Daily Living; SD, Standard Deviation; r, correlation coefficient; SOR, Self-Calculated Odds Ratio; BEHAVE-AD, Behavioural Pathology in Alzheimer’s disease RMBPC-NH, Revised Memory and Behaviour Problems Checklist-Nursing Home; DQoL, Dementia Quality of life; DS-DAT, Discomfort Scale - Dementia of Alzheimer Type; GLDS, Geriatric Level of Dysfunction Scale; rc/al, residential care/assisted living; MMSE, Mini Mental State Examination; OR, Odds Ratio.

**Table 6:** Correlates of pain with physical function

Correlates of pain and ADL or IADL					
*First author*	*N*	*Pain: prevalence*	*Physical function: prevalence*	*Correlates of pain with ADL or IADL*	*Quality of study*
**Brummel-Smith 2002** **[** 36 **]**	104 (excluding those unable to self-report pain)	Moderate-severe pain 60%, no-mild pain 40% (50 subject unable to answer)	≥1 ADL limitations 92% in dementia sample (n=154)	SOR 1.9 (95% CI: 0.0) with ≥ 1 ADL limitation	7
**Cipher 2004** **[** 4 **]**	234	Persistent pain 72%	ADL independency mean 0.09 (SD 0.99)	*Correlations with GMPI ‘pain and suffering’* r=−0.04 (α>0.05) with ADL independency	7.5
**Shega 2005** **[** 44 **]**	115	Any current pain self-report 32%, caregiver report 53%	KATZ mean 8.5 (SD 2.7), IADL mean 15.3 (SD 3.9)	*For self-report pain* No association ADL and IADL (p> 0.05) *For caregiver pain* report No association with ADL or IADL (p> 0.05)	9.5
**Shega 2010** **[** 45 **]**	5549	Moderate or greater pain: 35.8%	Any IADL impairment: 665%	**OR=1.74 (95% CI: 1.15-2.62)** with any iADL impairment (Adjusted for demographics)	9
**Torvik 2010** **[** 48 **]**	106	Current pain in total group 55%, in cognitive impaired group 52%	Highly or moderate ADL dependent 36%	p=0.20 for current pain and ADL SOR=0.5 (95% CI: 0.2-1.2) for current pain and ADL high/medium v.s. low	6.5
**Tosato 2012** **[** 3 **]**	2822	Any pain 19% (moderate/severe/excruciating pain 13%)	No disability 8%, assistance required 43%, dependent 49%	SOR 1.0 (95% CI: 0.9-1.2) with ADL-dependent SOR 0.9 (95% CI: 0.75-1.09) with ADL assistance required (Adjusted for age, gender, country, cognitive impairment, number of diseases, ischemic heart disease, stroke, falls, communication problems, and a flare-up of a chronic or recurrent condition)	11.5
**Correlates of pain and other functional impairments**					
**Black 2006** **[** 39 **]**	123	Pain 63%	Nutrition/hydration problems total sample 85%	SOR 1.9 (95% CI: 0.7-5.3) with nutrition/hydration problems	6.5
**Brummel-Smith 2002** **[** 40 **]**	104 (excluding those unable to self-report pain)	Moderate-severe pain 60%, no-mild pain 40% (50 subject unable to answer)	≥ 1 ADL limitations 92% in dementia sample (n=154)	SOR 1.6 (95% CI: 0.2) with bladder incontinence	7
**D’Astolfo 2006** **[** 44 **]**	140	Pain 64% (musculoskeletal pain 40%)	Use of wheel chair 60% Requires assistance 34%	SOR 1.5 (95% CI: 0.7-3.0) with use of wheel chair or bedridden SOR 1.0 (95% CI: 0.5-2.0) with requires assistance (Analyses in sample of no dementia-severe dementia)	7
**Lin 2011** **[** 46 **]**	112	Observed pain 37% (PAINAD >=2)	Being restrained 46%; observed care activities: bathing 43%, assisted transfer 31%, self-transfer 26%	**OR=5.4 (95% CI: 2.3-12.5)** and **AOR=3.0 (95% CI: 1.0-8.7)** with being restrained **OR=23.4 (95% CI: 3.0-188)** and **AOR=19.2 (95% CI: 2.3-162)** with bathing **OR=29.7 (95% CI: 3.6-242**) and **AOR=11.3 (95% CI: 1.2-102)** with assisted transfer, both compared to self-transfer (Adjusted for gender, age, wound, restraint, tube present in body, recent fall, severity of dementia and type of activity)	12
**Williams 2005** **[** 43 **]**	331	Pain 21%, in nh 23%, in rc/al 20% *(self-report for subgroup MMSE>10 was higher: 39% and 25%)*	Low activity 47%, immobile 12% Low food intake 53% Low fluid intake 51%	OR=0.65 (95% CI: 0.38-1.11) and AOR=0.64 (95% CI: 0.37-1.10) with low activity OR=1.1 (95% CI: 0.49-2.29) and AOR=0.8 (95% CI: 0.37-1.69) with immobility OR=1.18 (95% CI: 0.64-2.17) and AOR=1.03 (95% CI: 0.56-1.87) with low food intake OR=1.20 (95% CI: 0.67-2.15) and AOR 1.14 (95% CI: 0.66-1.99) with low fluid intake (Adjusted for: sex, race, age, cognitive status, number of 10 comorbidities, impairments of 7 activities of daily living)	10

**Abbreviations:** SOR, Self-Calculated Odds Ratio; ADL, Activities of Daily Living; SD, Standard Deviation; r, correlation coefficient; GMPI, Geriatric Multidimensional Pain and Illness Inventory; PAINAD, Pain Assessment in Advanced Dementia; OR, Odds Ratio; AOR, Adjusted Odds Ratio; KATZ, Index of Independence in Activities of Daily Living; IADL, Instrumental Activities of Daily Living; nh, nursing home; rc/al, residential care/assisted living; MMSE, Mini Mental State Examination.

In total we found 81 associations expressed in either ORs or correlations. The prevalence rates of pain, NPS, and impairment of physical function ranged from 19-72% [3,4], 2-85% [37,39] and 12-92%, respectively [40,43,45].

Of the 22 included articles, the ORs could be extracted in six and the correlation coefficient in nine articles; in addition, we could calculate the SOR for the associations in ten articles.

### Pain and neuropsychiatric symptoms

The most commonly described associations were between pain and depression (Table 3), pain and agitation (Table 4), and pain and specified NPS (Table 5), such as a negative association between pain and wandering, resistance to care, physical and verbal abuse, and aberrant vocalizations [3,36-38].

**Figure 2:**
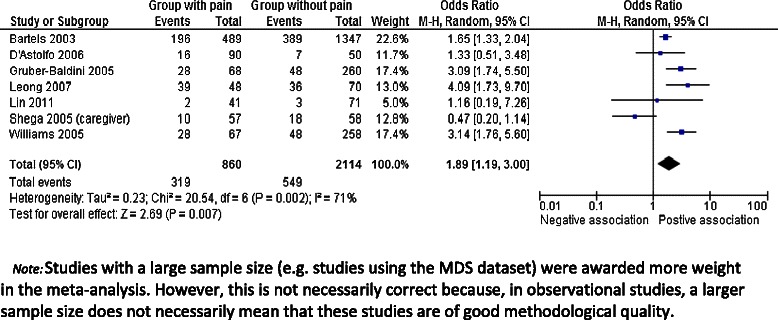
Forest plot: Pain and Depression.

Eleven articles described associations between pain and depression (Table 3); in seven of these there was a positive association, with three articles reporting a strong association with an OR >3 or = 0.5. In four articles the association was not significant: one article did not use a rating scale but examined medical records, one article used the rating scale PAINAD to measure pain, one article measured pain by observations, and another article used self-report. Remarkably, in the study by Shega et al. the OR for pain and depression was lower when pain was rated by the caregiver compared to the self-report of pain: OR 0.47 (95% CI: 0.20-1.14) and OR 1.52 (95% CI: 0.63-3.68), respectively [48]. We could include seven articles in the meta-analysis (see Figure 2) and the pooled OR for pain and depression was 1.84 (95% CI 1.23-2.80).

Eight articles described cross-sectional associations between pain and agitation/aggression (Table 4): four found positive associations, one found a negative association, two found no association, and one study found no association with pain self-report but a positive association with caregiver pain report. The strongest correlation found was in the study by Zieber et al., i.e. r = 0.51 (p < 0.01) between the DS-DAT scores and agitation.

Interestingly, two articles reported on longitudinal changes with follow-up data. In veterans living at home without aggressive behaviour in the preceding year or in the first five months of follow-up, Morgan et al. found that depression indirectly predicted the onset of aggression through pain [47]. In an unselected population Volicer et al. found that changes in agitation scores were related to changes in depression score but not to pain [51].

**Figure 3:**
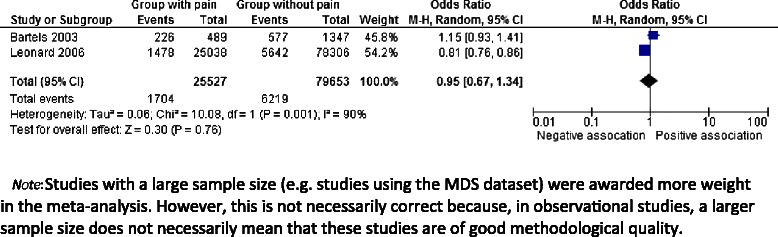
Forest plot: Pain and Agitation/Aggression.

Furthermore, in a subsample of patients with moderate dementia without the use of psychotropic medication, the association between pain and agitation/aggression was similar compared to residents who used psychotropic drugs [36]. Only two articles could be incorporated in the meta-analysis (see Figure 3) resulting in a pooled OR of 0.95 (95% CI 0.67-1.34).

Table 5 describes NPS, other than depression and agitation/aggression. Relations between pain and anxiety, hallucinations and delusions, were rarely studied. Only one article described an association between pain and anxiety, which was positive: SOR 1.8 (95% CI 1.0-3.0) [35]. Two articles described psychosis and delusions as being related to pain [3,34]. Kunik et al. found a small but non-significant association (r = 0.15; p >0.05) with psychosis and Tosato et al. found an OR of 1.5 (95% CI 1.07-2.03) between pain and delusions.

Furthermore, terms like ‘behavioural/psychiatric problems’ and ‘disruptive behaviour’ were also frequently used to describe unspecified NPS (Table 5). Two out of seven articles reported moderate positive associations, with r = 0.22 (p <0.05) as the strongest correlation between pain and dysfunctional behaviour [4].

### Pain and physical function

Eleven articles reported associations between pain and physical function, although in most cases this was not the main topic of the study (Table 6). We found associations between pain and ADL or iADL impairment [3,4,40,48,49,52]. One article reported a positive association between pain and iADL impairment: OR 1.74 (95% CI 1.15-2.62) [49]. Other associations (although not significant) with physical impairment described in the articles were immobility [44,46] and malnourishment [43].

Only two articles described a positive association: one study used the PAINAD to objectify pain and one study used a five-point verbal descriptive scale to measure pain and a three-point scale (OARS/IADL) to measure functional impairment [46,49].

**Figure 4:**
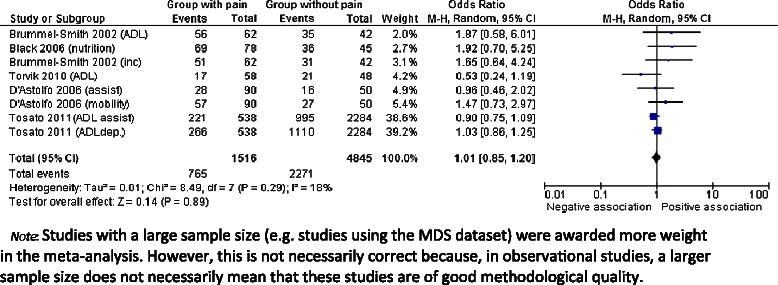
Forest plot: Pain and Physical Function (with reports of 5 out of 10 included studies).

The strongest reported association was with assisted transfer compared to self-transfer; however, this had a very broad confidence interval: OR 29.7 (95% CI 3.6-242) [46]. The remaining eight articles reported associations which were not significant. Based on five articles, the pooled OR (see Figure 4) for pain and overall physical function was 1.01 (95% CI 0.85-1.20).

## Discussion

Despite the increased attention for pain in dementia, relatively few studies have explored associations between pain and NPS, and pain and physical function. We found 22 articles reporting the strength of associations between these three modalities, including only two longitudinal studies.

We found most evidence for the association between pain and depression (in 7 of 11 articles), followed by the association between pain and agitation/aggression (in 5 of 8 articles). The two longitudinal studies reported no direct effects between pain and NPS but only some indirect effects, e.g. of pain through depression. Interestingly, articles reporting a significant positive association between pain and NPS, and between pain and physical function, were mainly of low methodological quality. One article with high methodological quality reported a non-significant correlation between pain frequency and verbal abuse [37]. Four high-quality articles reported a positive association between pain, aggression/agitation and wandering [36,51], between pain and functional impairment [46], and between pain and behavioural symptoms [43].

Due to the hypothesized effect of pain on NPS and physical function, and some overlap of items in the measurement instruments, we expected to find stronger associations; particularly since pain interventions targeting NPS and behavioural interventions targeting pain are reported to reduce both pain and NPS (such as depression and agitation/aggression) [54]. In addition, a cluster RCT by Husebo et al., investigating a sample of moderate to severe dementia patients with challenging behaviour, showed that treating pain led to a significant improvement in mood symptoms such as depression, apathy, and eating disorders, and improvements in ADL function were also found [12]. Furthermore, research among elderly without cognitive impairment shows an association between pain and depression; there is also evidence that treatment of depression in cognitively intact older patients improves pain and physical function [46,55,56]. It is plausible that this also applies to patients with dementia.

However, the associations found in the present systematic review were rather weak. This may be the result of inadequate assessment of both pain and NPS in the included studies. Most studies did not use measurement instruments developed for the assessment of pain in people with dementia. For example, D’Astolfo et al. did not use a measurement instrument for pain or for NPS, but only screened medical records and found relatively weak and non-significant associations. Also, it is possible that healthcare workers interpret NPS as symptoms of either pain *or* challenging behaviour; if this is the case, then only pain *or* NPS is reported in the medical records and no association will be found.

Five articles used the MDS-RAI Dataset to measure pain and also reported weak associations [3,36,37,50,51]. These articles also report weak associations. This might be due to the doubt about the accuracy of measuring pain in people suffering from dementia with the MDS-RAI Dataset [57,58].

We hypothesize that validated rating scales, used by a professional, will provide a more accurate reflection of the relationship between pain and NPS. This is illustrated by the study of Zieber et al. in which a clear distinction is seen in the strength of the correlations between pain and agitation when rated by a palliative nurse consultant or when rated by the facility nurse [38]. When rated by the palliative nurse consultant the correlation was stronger: = 0.49 (p < 0.01) compared with the rating by the facility nurse: r = 0.28 (p < 0.05). This also applied to the correlation between pain and aberrant vocalizations: r < 0.40 (p < 0.01) and r = 0.065 (p < 0.63), respectively, but not between pain and resisting care: r < 0.51 (p < 0.01) and r = 0.48 (p < 0.01), respectively. In addition, in a study by Leong et al. a professional used the PAINAD to asses pain and found a SOR of 3.2 (95% CI 1.8-5.9) between pain and depression [35]. However, other studies with a relative strong association between pain and depression did not use professionals or validated rating scales to assess pain in patients with dementia [43,45]. Therefore, the results of the present review cannot fully support the hypothesis of a better reflection of the relationship between pain and NPS when validated rating scales are used by professionals.

Another explanation for the rather weak associations found in this review could be the inclusion of six articles which described individuals with predominantly severe dementia. Together with the progression of dementia, the assessment of pain becomes even more difficult due to diminished pain behaviours [59], but facial expressions tend to increase in the course of dementia [60]. Of the measurement instruments used in the included studies, only the PAINAD and DS-DAT include facial expressions of pain. In addition, in the included studies, the use of antipsychotic drugs could also explain the weak associations. Antipsychotic drugs may distort and diminish the expression of NPS while a possible cause of NPS, for instance pain, is not treated. This may have resulted in the under-recognition and poor report of NPS. However, the study by Ahn et al. shows that, in a subsample of patients without psychotropic drugs, the association between pain and agitation/aggression, and between pain and wandering, was similar to that in residents who used psychotropic drugs [36].

Moreover, we could have anticipated finding rather weak associations, because most of the included studies were cross-sectional in design. This is illustrated by studies that found that a change in pain after an intervention is related to a decrease in NPS or function [61,62].

To some extent the included articles measured overall functional impairment with, for example, total ADL scores. Some articles focused on specific components of physical function, like nutritional status and mobility, which are often hampered in patients with dementia. However, because the focus of these articles was not on the association between pain and physical function, in most cases we had to calculate the association between pain and physical function (SOR) ourselves. This raises the question as to whether physical function is receiving the attention it deserves and, possibly, may even lead to publication bias. Physical inactivity or impairment is an important sign that a patient with dementia could be in pain; this is illustrated by a study in which patients with moderate to severe dementia (treated with acetaminophen) tend to spend more time in social interaction and engage with the environment more actively, than patients who received placebo [62]. Unfortunately, until now, no longitudinal studies are available that describe the course of physical function in patients with dementia in relation to pain.

### Strengths and limitations

This study is the first to give a comprehensive and systematic analysis of the associations between pain and NPS, and pain and physical function, in patients with dementia. One of the strengths of this study is that we not only included publications that presented associations between pain and NPS and pain and physical function, but also publications that provide enough information to compute ORs, thus taking full advantage of the available evidence. In addition, when possible, we present the crude OR as this reflects the presence of co-occurrence as perceived by the caregivers. Furthermore, we used a methodological quality assessment based on previously developed checklists [32,33]. By adding extra items focusing on the measurement of pain, study objective and population, we tailored the quality assessment to the purpose of this review. We believe that this strategy has led to a better reflection of the challenges in the assessment of pain and NPS.

A possible limitation could be some publication bias, e.g. if some studies do not report the associations because they were negative. Also, we explicitly searched for publications about pain and not for terms like ‘distress’ or ‘discomfort’. However, we believe that this approach provides the best reflection of the complex relation between pain, NPS and physical function. Furthermore, we were unable to include every study in the meta-analysis due to missing data. In addition, the forest plots should be interpreted with caution, since the included studies are heterogeneous and studies with a large sample size (e.g. studies using the MDS Dataset) were awarded more weight in the meta-analysis; however, this weighting is not necessarily justified because, in observational studies, a larger sample size does not necessarily means that these studies are of good methodological quality. Another possible limitation is that we did not include delirium as a separate search term in our search strategy. However, as delirium is a syndrome with specific neuropsychiatric symptoms, we looked at the clinical features of a delirium by including these symptoms, such as hallucinations and delusions, in our search strategy.

### Clinical implications

The American Geriatrics Society (AGS) published clinical guidance on persistent pain, outlining 26 behavioural expressions of pain in the elderly [21]. The AGS panel advises clinicians to assess pain in older persons with moderate to severe dementia via direct observation of this pain-related behaviour, or via history from caregivers. Several observational scales are available based on the presence of or alterations in behaviours, emotions, interactions, and facial expressions. However, there is little empirical evidence that these 26 behavioural expressions are indeed related to pain. In our review, only depression and agitation/aggression seem to be associated with pain.

The advice of direct observation of pain-related behaviour seems to be poorly implemented, as illustrated by this review, in which only three studies used rating scales based on behavioural observations [35,38,46]. It can be assumed that, when this non-optimal situation exists in a research setting, then routine implementation of rating scales based on behavioural observation in clinical practice will be even less optimal.

The results presented in this review do not fully support the association between pain, NPS and functional impairment in dementia. However, they do highlight the presence of difficulties in the management of pain in dementia. This is illustrated by the frequent use of terms like ‘behavioural symptoms’, ‘disruptive behaviour’, and ‘psychiatric symptoms’. There is no uniform way of reporting neuropsychiatric symptoms; this could complicate the comparison between behavioural symptoms and also reveals the challenges in differentiating between the different, but often very similar, types of challenging behaviour. This also applies to the description of physical function; the specific functions and activities should be properly described (e.g. malnutrition, sleep disturbances, and immobility) and not merely presented as a total ADL score.

Clearly, co-occurrence will not (and can not) be easily observed, probably leading to clinical indecisiveness. However, regardless of co-occurrence, we want to stress the importance of pain detection in patients with dementia because pain can be the cause of other disorders, such as NPS. Moreover, it has been proven that pain treatment significantly reduces behavioural disturbances, such as agitation [12,54,61]. Pain and it’s consequences have an impact on the quality of life and therefore should be recognized, measured and treated.

## Conclusions

This review shows, unexpectedly, rather weak associations between pain and NPS, and between pain and physical function. Nevertheless, the relationship between pain and the onset of NPS, as well as the effect on physical function, remains unclear and should be further explored. To unravel this complex relationship, the course of pain, NPS and physical function should be examined longitudinally, using valid measurement instruments. A longitudinal study design will provide more information on causality and the sequence of these modalities, providing evidence that can be incorporated in clinical practice to improve the management of pain for people with dementia.

## Additional files

Additional file 1: PRISMA 2009 checklist.
Additional file 2: Quality assessment of longitudinal studies.
Additional file 3: Rating scales for pain that were used in the reported studies.
