# Erratum to: Association between pain, neuropsychiatric symptoms, and physical function in dementia: a systematic review and meta-analysis

**DOI:** 10.1186/s12877-015-0085-1

**Published:** 2015-09-09

**Authors:** Annelore H. van Dalen-Kok, Marjoleine Pieper, Margot de Waal, Albert Lukas, Bettina S. Husebo, Wilco P. Achterberg

**Affiliations:** Department of Public Health and Primary Care, Leiden University Medical Centre, Hippocratespad 21 Post zone V0-P, PO Box 9600, Leiden, RC 2300 The Netherlands; Department of General Practice & Elderly Care Medicine, VU University Medical Centre Amsterdam, van der Boechorststraat 7, Amsterdam, BT 1081 The Netherlands; Malteser Hospital Bonn/Rhein-Sieg, Centre of Geriatric Medicine, Academic Hospital University of Bonn, Von-Hompesch-Straße 1, Bonn, 53123 Germany; Department of Public Health and Primary Care, Centre for Elderly and Nursing Home Medicine, University of Bergen, Bergen, Norway; Stavanger University Hospital, Bergen, Norway

The original version of this article unfortunately contained some mistakes. The presentation of Table 2, Table 5 and Table 6 was incorrect. The corrected tables are given below.

**Table 2:** Measurements of pain, neuropsychiatric symptoms and physical function

	Measurement of pain		Measurement of neuropsychiatric symptoms		Measurement of function	
First author	Rating scale	Method of detection	Rating scale	Method of detection	Rating scale	Method of detection
Ahn 2013^36^	MDS pain severity scale, combining pain frequency and pain intensity	Self-report, if not possible staff report based on proxy reports	MDS subscales; wandering-item, aggression behaviour scale (ABS), challenging behaviour profile (CBP) agitation subscale	Patient self-report, proxy and professional	MDS-ADL long form (7 items)	Staff observation
Bartels 2003^8^	No use of rating scale	Data collection instrument (3-month period), raters unknown	MDS for depression	Medical records	MDS (number of ADLs)	Medical records
Black 2006^39^	No use of rating scale	Medical records, preceding 6 months, interview surrogate and physician	No use of rating scales	Medical records, preceding 6 months, interview proxy and staff	No use of rating scale	Medical records, preceding 6 months, interview proxy and staff
Brummel-Smith 2002^40^	1 out of 3 scales: faces or line scale, or word-based pain intensity scale	self-report, assessed by trained research assistants	No use of rating scales	Trained research assistants	No use of rating scale	Trained research assistants
Cipher 2004^4^	GMPI pain and suffering subscale	Part of neuropsychological evaluation by a licensed clinical geropsychologist	-GDS-15 “-26 dysfunctional behaviours with scores “1-7”	Part of neuropsychological evaluation by a licensed clinical geropsychologist	PRADLI	Part of neuropsychological evaluation by a licensed clinical geropsychologist
Cipher 2006^41^	GMPI	Part of neuropsychological evaluation by a licensed clinical geropsychologist and each instrument was administered after interviewing the resident, nursing staff and family members	GLDS, 19 categories with scores 1-7	Part of neuropsychological evaluation by a licensed clinical geropsychologist and each instrument was administered after interviewing the resident, nursing staff and family members, Medical records, preceding 6 to max 26, Months	GLDS	Part of neuropsychological evaluation by a licensed clinical geropsychologist and each instrument was administered after interviewing the resident, nursing staff and family members
D’Astolfo 2006^44^	No use of rating scale	Medical records, preceding 6 to max 26 months	No use of rating scales		No use of rating scale	Medical records Ambulatory status: independent, requires assistance, wheel chair (or bedridden n?=?1)
Gruber-Baldini 2005^45^	PGC-PIS, score ≥ 2	Rating by supervisory staff member	CSDD	Rating by supervisory staff member	MDS; activities of daily living scale, SMOI	Rating/observation by supervisory staff member
			CMAI			
Kunik 2005^30^	PGC-PIS, item on level of pain in previous week, scores 1-6	Interview with patient and proxy by trained interviewer/research assistant	CMAI	Interview with patient and proxy by trained interviewer/research assistant	-	-
			HAM-D			
			NPI (subdomains delusion/hallucinations)			
Leonard 2006^50^	MDS pain burden using a 4-level composite score based on pain frequency and intensity	-	MDS (Physical aggression: MDS item 'others were hit, shoved, scratched, sexually abused'; Depression: MDS score ≥3 on sum of 9 items, e.g. 'being sad', 'making negative statements', 'persistent anger with self or others', 'pained facial expressions'. (At least once in week before)	-	-	-
Leong 2007^35^	PAINAD for non-communicative patients	Interviews with patient and staff member by professionals for communicative patients	Depression with GDS-15 or STAI	Self-report or staff report	AAS	Not reported
			Anxiety with Cornell			
Lin 2011^46^	PAINAD-Chinese version	Observation immediately following instances of routine care by principal investigator and research assistant	No use of rating scales	Medical records and observations by professional	No use of rating scale	Medical records and observation by professional
Morgan 2012^47^	PGC-PIS worst pain item	Not reported	CMAI aggression subscale	Not reported	-	-
			CMAI non-aggressive physical agitation subscale			
			HAM-D depression			
Norton 2010^42^	PPQ, intensity item, 10–14 day baseline	Primary CNA and data used from medical records	RMBPC-NH, selection of 3 need driven behaviours, BEHAVE-AD	Primary CNA and unit staff	PSMS	Nurses and trained research assistants
Shega 2005^48^	VDS, 1 item on presence and severity of pain ‘right now’	Interviews with patients and caregivers by trained research assistant	GDS-15	Interview patient and proxy	KATZ	Interview patient and proxy
			CMAI		IADL	
Shega 2010^49^	VDS, 5 point, ‘pain past 4 weeks’	Interviews with patient by trained research assistant	Mental Health screening questionnaire; 5-item and 6 point scale	Interview with patient by trained research assistant	OARS/IADL; 3 point scale	Interview patient by trained research assistant
Torvik 2010^48^	VRS, 4 point, ‘pain right now’	Patient self-report	DQoL, 29-items on 5 domains: self-esteem, aesthetics, positive affect, negative affect, belonging	Not reported	Barthel	Self-report and medical records
Tosato 2012^3^	InterRAI LTCF	InterRAI LTCF questions and observation of behaviour, any type of pain or discomfort of the body in previous 3 days by trained (research) staff	InterRAI LTCF 5 behavioural symptoms, previous 3 days	Not reported	MDS ADL Hierarchy Scale	Data recorded by study physicians
Volicer 2009^37^	MDS-RAI pain frequency (item J2a)	Combination of physical examination, patient history, observation, consultation caregiver and medical records by staff	MDS Depression Rating Scale	Combination of physical examination, patient history, observation, consultation caregiver and medical records by staff	-	-
			MDS item J1e for delusions MDS item J1i for hallucinations			
Volicer 2011^51^	MDS	Combination of physical examination, patient history, observation, consultation caregiver and medical records by staff	MDS items I1ee, E1a, E1d, E1f, E1b, E1i, E1l, E1m for depression	Combination of physical examination, patient history, observation, consultation caregiver and medical records by staff	-	-
			MDS for delusions and hallucinations			
			MDS items B5b, E1b, E4aa, E4da for agitation			
Williams 2005^43^	PGC-PIS, score =2, and 0–10 pain numeric rating scale	Registered nurses or licensed practical nurses and interview with overseeing supervisor	CSDD, score =7	Rating by care supervisors, registered nurses and licensed practical nurses	MDS-ADL	Rating by care supervisors, registered nurses and licensed practical nurses
			CMAI, any behaviour at least weekly		APAS	
					SMOI	
Zieber 2005^38^	DS-DAT, and a 7-point pain rating scale	Trained facility nurses, palliative care nurse consultants	PAS	Trained facility nurses	-	-

*Abbreviations: MDS* Minimum Dataset, *ADL* Activities of Daily Living, *GMPI* Geriatric Multidimensional Pain and Illness Inventory, *GDS-15* Geriatric Depression Scale-15 short version, *PRADLI* Psychosocial Resistance to Activities of Daily Living Index, *GLDS* Geriatric Level of Dysfunction Scale, *PGC-PIS* Philadelphia Geriatric Centre Pain Intensity Scale, *CSDD* Cornell Scale for Depression in Dementia, *CMAI* Cohen-Mansfield Agitation Inventory, *SMOI* Structured Meal Observational Instrument, *HAM-D* Hamilton Rating Scale for Depression, *NPI* Neuropsychiatric Inventory, *PAINAD* Pain Assessment in Advanced Dementia, *STAI* State-Trait Anxiety Inventory, *AAS* Adjusted Activity Scale, *PPQ* Proxy Pain Questionnaire, *CNA* Certified Nursing Assistant, *RMBPC-NH* Revised Memory and Behaviour Problems Checklist-Nursing Home, *BEHAVE-AD* Behavioural Pathology in Alzheimer’s disease, *PSMS* Physical Self Maintenance Scale, *VDS* Verbal Descriptor Scale, *KATZ* Index of Independence in Activities of Daily Living, *IADL* Instrumental Activities of Daily Living, *OARS/IADL* Older Americans Recourses and Services/Instrumental Activities of Daily Living, *VRS* Verbal Rating Scale, *DQol* Dementia Quality of life, *APAS* Albert Patient activity Scale, *DS-DAT* Discomfort Scale - Dementia of Alzheimer Type, *PAS* Pittsburgh Agitation Scale

**Table 5:** Correlates of pain and neuropsychiatric symptoms

Correlates of pain and specified NPS					
First author	N	Pain: prevalence	Neuropsychiatric symptoms: prevalence	Correlates of pain with NPS	Quality of study
Ahn 2013^36^	56577	Not reported	Wandering 9 %	AOR 0.77 (95 % CI: 0.73-0.81) with wandering	10
				Subsample without psychotropic medication	
				AOR 0.72 (95 % CI: 0.63-0.83) with wandering	
				(Adjusted for cognition, ADL, sociodemographics)	
Kunik 2005^34^	99	Pain mean 2.4 (SD 1.2)	Delusions/hallucinations mean 0.35 (SD 0.48)	r = 0.15 (p > 0.05) with psychosis	8.5
Leong 2007^35^	225	Pain 44 %, chronic pain 34 %	Anxiety 48 %	SOR 1.8 (95 % CI: 1.0-3.0) with anxiety	8.5
Norton 2010^42^	161	Not reported	BEHAVE-AD mean 6..4 (SD 29.2)	r = 0.15 (p = 0.08) for pain intensity and emotional behaviour problems	9
			RMBPC-NH mean 1.45 (SD 0.64)	r = 0.05 (p = 0.58) for pain intensity and resistiveness to care	
Torvik 2010^52^	106	Current pain in total group 55 %, in cognitive impaired group 52 %	Negative affect index (DQoL) mean 2.0 (SD 0.75), positive affect/humour index (DQoL) mean 3.4 (SD 0.9)	p < 0.01 for current pain and negative affect	6.5
				p = 0.11 for current pain and with positive affect/humour	
Tosato 2012^3^	2822	Any pain 19 % (moderate/severe/excruciating pain 13 %)	Behavioural symptoms 37 % Psychiatric symptoms 21 %	AOR = 0.74 (95 % CI: 0.55-1.0) with wandering	11.5
				AOR = 1.4 (95 % CI: 1.08-1.8) with resistance to care	
				AOR 1.5 (95 % CI: 1.07-2.03) with delusions	
				AOR 1.06 (95 % CI: 0.80-1.41) with verbal abuse	
				AOR 1.08 (95 % CI: 0.75-1.55) with physical abuse	
				(Adjusted for age, gender, country, cognitive impairment, number of diseases, ischemic heart disease, stroke, falls, communication problems, and a flare-up of a chronic or recurrent condition)	
Volicer 2009^37^	929	Daily pain 29 %, less than daily pain 19 %	Verbally abusive not easily altered 2 %, physically abusive not easily altered 12 %	r = 0.07 (p = 0.03) for pain frequency and verbal abuse	11
				AOR = 0.9 (p = 0.53) with resisting care	
				AOR = 0.7 (p = 1.2) with verbal abuse	
				AOR = 0.7 (p = 0.16) with physical abuse	
			Delusions 8 %	(Both multivariate models among others controlled for resisting care)	
			Hallucinations 9 %		
Zieber 2005^38^	58	Not reported	Not reported	r = 0.46 (p < 0.01) for DS-DAT scores and resisting care	8
				r = 0.42 (p < 0.01) for DS-DAT scores and aberrant vocalization	
				Pain rating by palliative care nurse consultants:	
				r = 0.51 (p < 0.01) with resisting care	
				r = 0.40 (p < 0.01) with aberrant vocalizations	
				Pain rating by facility nurse:	
				r = 0.48 (p < 0.01) with resisting care	
				r = 0.065 (p < 0.63) with aberrant vocalizations	
Correlates of pain and unspecified NPS					
First author	N	Pain: prevalence	Neuropsychiatric symptoms: prevalence	Correlates of pain with unspecified NPS	Quality of study
Black 2006^39^	123	Pain 63 %	Psychiatric disorders or behaviour problems 85 %, behaviour problems 67 %	SOR 1.9 (95 % CI: 0.7-5.3) with psychiatric/behaviour problems	6.5
				SOR 1.2 (95 % CI: 0.5-2.5) with behaviour problems	
Brummel-Smith 2002^40^	104 (excluding those unable to self-report pain)	Moderate-severe pain 60 %	≥1 disruptive behaviours (wandering, verbal disruption, physical aggression, regressive behaviour, hallucinations)	SOR 1.8 (95 % CI: 0.8-4.0) with ≥ 1 disruptive behaviour	7
		No-mild pain 40 %			
		50 subject unable to answer			
			70 % in dementia sample n = 154		
Cipher 2004^4^	234	Persistent pain 72 %	Dysfunctional behaviours mean 4.4 (SD 0.76)	r = 0.22 (p < 0.05) with dysfunctional behaviours	7.5
Cipher 2006^41^	277	Acute pain 29 %	-	r = 0.18 (p < 0.05) with GLDS mean behavioural intensity	7.5
		Chronic pain 59 %			
Norton 2010^42^	161	Not reported	BEHAVE-AD mean 61.4 (SD 29.2)	r = 0.18 (p = 0.03) for pain intensity and disruptive behaviour problems	9
			RMBPC-NH mean 1.45 (SD 0.64)	r = 0.05 (p = 0.53) for pain intensity and global need driven behaviours	
Tosato 2012^3^	2822	Any pain 19 % (moderate/severe/excruciating pain 13 %)	Behavioural symptoms 37 %	AOR = 1.4 (95 % CI: 1.04-1.8) with socially inappropriate behaviour	11.5
			Psychiatric symptoms 21 %	(Adjusted for age, gender, country, cognitive impairment, number of diseases, ischemic heart disease, stroke, falls, communication problems, and a flare-up of a chronic or recurrent condition)	
Williams 2005^39^	331	Pain 21 %, in nh 23 %, in rc/al 20 % (self-report for subgroup mmse > 10 was higher: 39 % and 25 %)	Behavioural symptoms 58 %	OR = 1.1 (95 % CI: 0.49-2.29) and AOR = 1.2 (95 % CI: 0.57-2.36) with behavioural symptoms	10
				(Adjusted for: sex, race, age, cognitive status, number of 10 comorbidities, impairments of 7 activities of daily living)	

*Abbreviations: AOR* Adjusted Odds Ratio, *ADL* Activities of Daily Living, *SD* Standard Deviation, *r* correlation coefficient, *SOR* Self-Calculated Odds Ratio, *BEHAVE-AD* Behavioural Pathology in Alzheimer’s disease, *RMBPC-NH* Revised Memory and Behaviour Problems Checklist-Nursing Home, *DQoL* Dementia Quality of life, *DS-DAT* Discomfort Scale - Dementia of Alzheimer Type, *GLDS* Geriatric Level of Dysfunction Scale, *rc/al* residential care/assisted living, *MMSE* Mini Mental State Examination, *OR* Odds Ratio

**Table 6:** Correlates of pain with physical function

Correlates of pain and ADL or IADL					
First author	N	Pain: prevalence	Physical function: prevalence	Correlates of pain with ADL or IADL	Quality of study
Brummel-Smith 2002^36^	104 (excluding those unable to self-report pain)	Moderate-severe pain 60 %, no-mild pain 40 % (50 subject unable to answer)	≥1 ADL limitations	SOR 1.9 (95 % CI: 0.6-6.0) with ≥ 1 ADL limitation	7
			92 % in dementia sample (n = 154)		
Cipher 2004^4^	234	Persistent pain 72 %	ADL independency mean 0.09 (SD 0.99)	Correlations with GMPI ’pain and suffering’	7.5
				r = −0.04 (α > 0.05) with ADL independency	
Shega 2005^44^	115	Any current pain self-report 32 %, caregiver report 53 %	KATZ mean 8.5 (SD 2.7), IADL mean 15.3 (SD 3.9)	For self-report pain	9.5
				No association ADL and IADL (p > 0.05)	
				For caregiver pain report	
				No association with ADL or IADL (p > 0.05)	
Shega 2010^45^	5549	Moderate or greater pain: 35.8 %	Any IADL impairment: 66.5 %	OR = 1.74 (95 % CI: 1.15-2.62) with any iADL impairment	9
				(Adjusted for demographics)	
Torvik 2010^48^	106	Current pain in total group 55 %, in cognitive impaired group 52 %	Highly or moderate ADL dependent 36 %	p = 0.20 for current pain and ADL	6.5
				SOR = 0.5 (95 % CI: 0.2-1.2) for current pain and ADL high/medium v.s. low	
Tosato 2012^3^	2822	Any pain 19 % (moderate/severe/excruciating pain 13 %)	No disability 8 %, assistance required 43 %, dependent 49 %	SOR 1.0 (95 % CI: 0.9-1.2) with ADL-dependent	11.5
				SOR 0.9 (95 % CI: 0.75-1.09) with ADL assistance required	
				(Adjusted for age, gender, country, cognitive impairment, number of diseases, ischemic heart disease, stroke, falls, communication problems, and a flare-up of a chronic or recurrent condition)	
Correlates of pain and other functional impairments					
First author	N	Pain: prevalence	Physical function: prevalence	Correlates of pain with ADL or IADL	Quality of study
Black 2006^39^	123	Pain 63 %	Nutrition/hydration problems total sample 85 %	SOR 1.9 (95 % CI: 0.7-5.3) with nutrition/hydration problems	6.5
Brummel-Smith 2002^40^	104 (excluding those unable to self-report pain)	Moderate-severe pain 60 %, no-mild pain 40 % (50 subject unable to answer)	≥1 ADL limitations	SOR 1.6 (95 % CI: 0.6-4.2) with bladder incontinence	7
			92 % in dementia sample (n = 154)		
D’Astolfo 2006^44^	140	Pain 64 % (musculoskeletal pain 40 %)	Use of wheel chair 60 %	SOR 1.5 (95 % CI: 0.7-3.0) with use of wheel chair or bedridden	7
			Requires assistance 34 %	SOR 1.0 (95 % CI: 0.5-2.0) with requires assistance	
				(Analyses in sample of no dementia-severe dementia)	
Lin 2011^46^	112	Observed pain 37 % (PAINAD > =2)	Being restrained 46 %; observed care activities: bathing 43 %, assisted transfer 31 %, self-transfer 26 %	OR = 5.4 (95 % CI: 2.3-12.5) and AOR = 3.0 (95 % CI: 1.0-8.7) with being restrained	12
				OR = 23.4 (95 % CI: 3.0-188) and AOR = 19.2 (95 % CI: 2.3-162) with bathing	
				OR = 29.7 (95 % CI: 3.6-242) and AOR = 11.3 (95 % CI: 1.2-102) with assisted transfer, both compared to self-transfer	
				(Adjusted for gender, age, wound, restraint, tube present in body, recent fall, severity of dementia and type of activity)	
Williams 2005^43^	331	Pain 21 %, in nh 23 %, in rc/al 20 % (self-report for subgroup MMSE > 10 was higher: 39 % and 25 %)	Low activity 47 %, immobile 12 %	OR = 0.65 (95 % CI: 0.38-1.11) and AOR = 0.64 (95 % CI: 0.37-1.10) with low activity	10
			Low food intake 53 %	OR = 1.1 (95 % CI: 0.49-2.29) and AOR = 0.8 (95 % CI: 0.37-1.69) with immobility	
			Low fluid intake 51 %	OR = 1.18 (95 % CI: 0.64-2.17) and AOR = 1.03 (95 % CI: 0.56-1.87) with low food intake	
				OR = 1.20 (95 % CI: 0.67-2.15) and AOR 1.14 (95 % CI: 0.66-1.99) with low fluid intake	
				(Adjusted for: sex, race, age, cognitive status, number of 10 comorbidities, impairments of 7 activities of daily living)	

*Abbreviations: SOR* Self-Calculated Odds Ratio, *ADL* Activities of Daily Living, *SD* Standard Deviation, *r* correlation coefficient, *GMPI* Geriatric Multidimensional Pain and Illness Inventory, *PAINAD* Pain Assessment in Advanced Dementia, *OR* Odds Ratio, *AOR* Adjusted Odds Ratio, *KATZ* Index of Independence in Activities of Daily Living, *IADL* Instrumental Activities of Daily Living, *nh* nursing home, *rc/al* residential care/assisted living, *MMSE* Mini Mental State Examination

