# Supplementary material for: Association between pain, neuropsychiatric symptoms, and physical function in dementia: a systematic review and meta-analysis
Source: BMC Geriatr. 2015 Apr 19;15:49. doi: 10.1186/s12877-015-0048-6 (PMC4409739; doi:10.1186/s12877-015-0048-6)
Supplement: Additional file 2: — Quality assessment of longitudinal studies. [file 12877_2015_48_MOESM2_ESM.doc]

**Additional file 2. Quality assessment of longitudinal studies**

| **Author** | Morgan[43] | Volicer[47] |
| --- | --- | --- |
| **Year** | 2021 | 2011 |
| **Journal** | Gerontologist | Int J Geriatr Psychiatry |
|  |  |  |
| ***Study population*** |  |  |
| Is there a specific, clearly stated objective described? | 1 | 1 |
| Is the stated objective similar to our objective? | 0 | 1 |
|  |  |  |
| ***Study population*** |  |  |
| Were valid selection criteria used for the study population? (sampling frame and distribution of the population by age and sex) | 1 | 1 |
| Is there a specified selected population? (other than severity of dementia and age)  For example a population selected on pain (e.g. pressure ulcers), behavior or function.(yes=o, no=1) | 0 | 1 |
| Did more than 80% of the eligible subjects participate in the study?  Or did 60-80% participate and non-responders were not selective? (data presented) | 1 | 1 |
| Is there major and selective loss during follow-up? (yes=0, no=1) | 1 | 1 |
|  |  |  |
| ***Measurement of pain*** |  |  |
| Was pain measured with a valid measurment instrument? (rating of observations (2), rating based on observations (1), self-report instruments (0,5) no measurement instrument (0)) | 1 | 1 |
| Was the method of detection by direct observation (1), interview staff/proxy/self-report (0,5) or screening medical records (0)? | 0.5 | 1.5^*^ |
| Was the measurement performed by a trained professional/research assistant (1), staff member (0,5) or a proxy/self-report (0)? | 0 | 0.5 |
|  |  |  |
| ***Outcome measure (behavior, physical function)*** |  |  |
| Was the outcome measured with a valid and reproducible method? | 1 | 1 |
| Was the follow-up period sufficient enough? | 1 | 1 |
|  |  |  |
| ***Analysis*** |  |  |
| Were the results adjusted for possible confounders? | 1 | 0 |
| Is there an association presented (OR/RR/correlation coefficient), including 95% CIs and numbers in the analyses? | 1 | 1 |
|  |  |  |
| Total score | 9.5 | 12 |

*Combination of observation and interview

**Additional file 3. Quality assessment of cross-sectional observation studies**

| **Author** | Ahn[32] | Bartels[8] | Black[35] | Brummel-Smith[36] | Cipher[4] | Cipher[37] |
| --- | --- | --- | --- | --- | --- | --- |
| **Year** | 2003 | 2003 | 2006 | 2002 | 2004 | 2006 |
| **Journal** | BMC Geriatrics | Am J Geriatr Psychiatry | Alzheimer Dis&Ass Dis | J Am Geriatr Soc | Int J Geriatr Psychiatry | J Am Med Dir Assoc |
|  |  |  |  |  |  |  |
| ***Study population*** |  |  |  |  |  |  |
| Is there a specific, clearly stated objective described? | 1 | 1 | 1 | 1 | 1 | 1 |
| Is the stated objective similar to our objective? | 1 | 0 | 1 | 0 | 0 | 1 |
|  |  |  |  |  |  |  |
| ***Study population*** |  |  |  |  |  |  |
| Were valid selection criteria used for the study population? (sampling frame and distribution of the population by age and sex) | 1 | 1 | 1 | 1 | 1 | 1 |
| Is there a specified selected population? (other than severity of dementia and age)  For example a population selected on pain (e.g. pressure ulcers), behavior or function.(yes=o, no=1) | 1 | 0 | 0 | 0 | 0 | 0 |
| Did more than 80% of the eligible subjects participate in the study?  Or did 60-80% participate and non-responders were not selective? (data presented) | 1 | 1 | 0 | 1 | 1 | 0 |
|  |  |  |  |  |  |  |
| ***Measurement of pain*** |  |  |  |  |  |  |
| Was pain measured with a valid measurment instrument? (rating of observations (2), rating based on observations (1), self-report instruments (0,5) no measurement instrument (0)) | 1 | 0 | 0 | 0.5 | 1 | 1 |
| Was the method of detection by direct observation (1), interview staff/proxy/self-report (0,5) or screening medical records (0)? | 0.5 | 0 | 0.5 | 0.5 | 0.5 | 0.5 |
| Was the measurement performed by a trained professional/research assistant (1), staff member (0,5) or a proxy/self-report (0)? | 0.5 | 0 | 0 | 1 | 1 | 1 |
|  |  |  |  |  |  |  |
| ***Outcome measure (behavior, physical function)*** |  |  |  |  |  |  |
| Was the outcome measured with a valid and reproducible method? | 1 | 1 | 1 | 1 | 1 | 1 |
|  |  |  |  |  |  |  |
| ***Analysis*** |  |  |  |  |  |  |
| Were the results adjusted for possible confounders? | 1 | 0 | 1 | 1 | 0 | 0 |
| Is there an association presented (OR/RR/correlation coefficient), including 95% CIs and numbers in the analyses? | 1 | 0 | 1 | 0 | 1 | 1 |
|  |  |  |  |  |  |  |
| Total score | 10 | 4 | 6.5 | 7 | 7.5 | 7.5 |

**Continued: Additional file 3. Quality assessment of cross-sectional observation** studies

| **Author** | D’Astolfo[40] | Gruber-Baldini[41] | Kunik[30] | Leonard[46] | Leong[31] | Lin[42] |
| --- | --- | --- | --- | --- | --- | --- |
| **Year** | 2006 | 2006 | 2005 | 2006 | 2007 | 2011 |
| **Journal** | BMC Geriatrics | Gerontologist | Psychiatr Serv | Arch Intern Med | Clin J Pain | Journal of Clinical Nurs |
|  |  |  |  |  |  |  |
| ***Study population*** |  |  |  |  |  |  |
| Is there a specific, clearly stated objective described? | 1 | 1 | 1 | 1 | 1 | 1 |
| Is the stated objective similar to our objective? | 1 | 0 | 1 | 1 | 0 | 1 |
|  |  |  |  |  |  |  |
| ***Study population*** |  |  |  |  |  |  |
| Were valid selection criteria used for the study population? (sampling frame and distribution of the population by age and sex) | 1 | 1 | 1 | 1 | 1 | 1 |
| Is there a specified selected population? (other than severity of dementia and age)  For example a population selected on pain (e.g. pressure ulcers), behavior or function.(yes=o, no=1) | 1 | 1 | 1 | 1 | 1 | 1 |
| Did more than 80% of the eligible subjects participate in the study?  Or did 60-80% participate and non-responders were not selective? (data presented) | 1 | 1 | 0 | 1 | 1 | 1 |
|  |  |  |  |  |  |  |
| ***Measurement of pain*** |  |  |  |  |  |  |
| Was pain measured with a valid measurment instrument? (rating of observations (2), rating based on observations (1), self-report instruments (0,5) no measurement instrument (0)) | 0 | 1 | 1 | 1 | 2 | 2 |
| Was the method of detection by direct observation (1), interview staff/proxy/self-report (0,5) or screening medical records (0)? | 0 | 0 | 0.5 | 0 | 0.5 | 1 |
| Was the measurement performed by a trained professional/research assistant (1), staff member (0,5) or a proxy/self-report (0)? | 0 | 0.5 | 1 | 0 | 1 | 1 |
|  |  |  |  |  |  |  |
| ***Outcome measure (behavior, physical function)*** |  |  |  |  |  |  |
| Was the outcome measured with a valid and reproducible method? | 1 | 1 | 1 | 1 | 1 | 1 |
|  |  |  |  |  |  |  |
| ***Analysis*** |  |  |  |  |  |  |
| Were the results adjusted for possible confounders? | 1 | 1 | 0 | 1 | 0 | 1 |
| Is there an association presented (OR/RR/correlation coefficient), including 95% CIs and numbers in the analyses? | 0 | 1 | 1 | 1 | 0 | 1 |
|  |  |  |  |  |  |  |
| Total score | 7 | 8.5 | 8.5 | 9 | 8.5 | 12 |

| **Author** | Norton[43] | Shega[44] | Shega[45] | Torvik[48] | Tosato[3] | Volicer[33] |
| --- | --- | --- | --- | --- | --- | --- |
| **Year** | 2010 | 2005 | 2010 | 2010 | 2012 | 2009 |
| **Journal** | Gerontologist | J Palliat Med | Pain Med | Pain Manag Nurs | Pain | J Am Med Dir Assoc |
|  |  |  |  |  |  |  |
| ***Study population*** |  |  |  |  |  |  |
| Is there a specific, clearly stated objective described? |  |  |  |  |  |  |
| Is the stated objective similar to our objective? |  |  |  |  |  |  |
|  |  |  |  |  |  |  |
| ***Study population*** |  |  |  |  |  |  |
| Were valid selection criteria used for the study population? (sampling frame and distribution of the population by age and sex) |  |  |  |  |  |  |
| Is there a specified selected population? (other than severity of dementia and age)  For example a population selected on pain (e.g. pressure ulcers), behavior or function.(yes=o, no=1) |  |  |  |  |  |  |
| Did more than 80% of the eligible subjects participate in the study?  Or did 60-80% participate and non-responders were not selective? (data presented) |  |  |  |  |  |  |
|  |  |  |  |  |  |  |
| ***Measurement of pain*** |  |  |  |  |  |  |
| Was pain measured with a valid measurment instrument? (rating of observations (2), rating based on observations (1), self-report instruments (0,5) no measurement instrument (0)) |  |  |  |  |  |  |
| Was the method of detection by direct observation (1), interview staff/proxy/self-report (0,5) or screening medical records (0)? |  |  |  |  |  |  |
| Was the measurement performed by a trained professional/research assistant (1), staff member (0,5) or a proxy/self-report (0)? |  |  |  |  |  |  |
|  |  |  |  |  |  |  |
| ***Outcome measure (behavior, physical function)*** |  |  |  |  |  |  |
| Was the outcome measured with a valid and reproducible method? |  |  |  |  |  |  |
|  |  |  |  |  |  |  |
| ***Analysis*** |  |  |  |  |  |  |
| Were the results adjusted for possible confounders? |  |  |  |  |  |  |
| Is there an association presented (OR/RR/correlation coefficient), including 95% CIs and numbers in the analyses? |  |  |  |  |  |  |
|  |  |  |  |  |  |  |
| Total score |  |  |  |  |  |  |

**Continued: Additional file 3. Quality assessment of cross-sectional observation** studies

| **Author** | Norton[43] | Shega[44] | Shega[45] | Torvik[48] | Tosato[3] | Volicer[33] |
| --- | --- | --- | --- | --- | --- | --- |
| **Year** | 2010 | 2005 | 2010 | 2010 | 2012 | 2009 |
| **Journal** | Gerontologist | J Palliat Med | Pain Med | Pain Manag Nurs | Pain | J Am Med Dir Assoc |
|  |  |  |  |  |  |  |
| ***Study population*** |  |  |  |  |  |  |
| Is there a specific, clearly stated objective described? | 1 | 1 | 1 | 1 | 1 | 1 |
| Is the stated objective similar to our objective? | 1 | 1 | 0 | 0 | 1 | 1 |
|  |  |  |  |  |  |  |
| ***Study population*** |  |  |  |  |  |  |
| Were valid selection criteria used for the study population? (sampling frame and distribution of the population by age and sex) | 1 | 1 | 1 | 1 | 1 | 1 |
| Is there a specified selected population? (other than severity of dementia and age)  For example a population selected on pain (e.g. pressure ulcers), behavior or function.(yes=o, no=1) | 0 | 1 | 1 | 0 | 1 | 1 |
| Did more than 80% of the eligible subjects participate in the study?  Or did 60-80% participate and non-responders were not selective? (data presented) | 1 | 1 | 1 | 0 | 1 | 1 |
| 1 |  |  |  |  |  |  |
| ***Measurement of pain*** |  |  |  |  |  |  |
| Was pain measured with a valid measurment instrument? (rating of observations (2), rating based on observations (1), self-report instruments (0,5) no measurement instrument (0)) | 1 | 1 | 0.5 | 1 | 1 | 1 |
| Was the method of detection by direct observation (1), interview staff/proxy/self-report (0,5) or screening medical records (0)? | 0.5 | 0.5 | 0.5 | 0.5 | 0.5 | 1 |
| Was the measurement performed by a trained professional/research assistant (1), staff member (0,5) or a proxy/self-report (0)? | 0.5 | 1 | 1 | 1 | 1 | 0 |
|  |  |  |  |  | 1 | 1 |
| ***Outcome measure (behavior, physical function)*** |  |  |  |  |  |  |
| Was the outcome measured with a valid and reproducible method? | 1 | 1 | 1 | 1 | 1 | 1 |
|  |  |  |  |  |  |  |
| ***Analysis*** |  |  |  |  |  |  |
| Were the results adjusted for possible confounders? | 1 | 0 | 1 | 0 | 1 | 1 |
| Is there an association presented (OR/RR/correlation coefficient), including 95% CIs and numbers in the analyses? | 1 | 1 | 1 | 1 | 1 | 1 |
|  |  |  |  |  |  |  |
| Total score | 9 | 9.5 | 9 | 6.5 | 11.5 | 11 |

**Continued:** **Additional file 3. Quality assessment of cross-sectional observation studies**

| **Author** | Williams[39] | Zieber[34] |
| --- | --- | --- |
| **Year** | 2005 | 2005 |
| **Journal** | Gerontologist | Int J Palliat Nurs |
|  |  |  |
| ***Study population*** |  |  |
| Is there a specific, clearly stated objective described? | 1 | 1 |
| Is the stated objective similar to our objective? | 1 | 1 |
|  |  |  |
| ***Study population*** |  |  |
| Were valid selection criteria used for the study population? (sampling frame and distribution of the population by age and sex) | 1 | 1 |
| Is there a specified selected population? (other than severity of dementia and age)  For example a population selected on pain (e.g. pressure ulcers), behavior or function.(yes=o, no=1) | 1 | 0 |
| Did more than 80% of the eligible subjects participate in the study?  Or did 60-80% participate and non-responders were not selective? (data presented) | 1 | 0 |
|  |  |  |
| ***Measurement of pain*** |  |  |
| Was pain measured with a valid measurment instrument? (rating of observations (2), rating based on observations (1), self-report instruments (0,5) no measurement instrument (0)) | 1 | 2 |
| Was the method of detection by direct observation (1), interview staff/proxy/self-report (0,5) or screening medical records (0)? | 0.5 | 1 |
| Was the measurement performed by a trained professional/research assistant (1), staff member (0,5) or a proxy/self-report (0)? | 0.5 | 1 |
|  |  |  |
| ***Outcome measure (behavior, physical function)*** |  |  |
| Was the outcome measured with a valid and reproducible method? | 1 | 0 |
|  |  |  |
| ***Analysis*** |  |  |
| Were the results adjusted for possible confounders? | 1 | 0 |
| Is there an association presented (OR/RR/correlation coefficient), including 95% CIs and numbers in the analyses? | 1 | 1 |
|  |  |  |
| Total score | 10 | 8 |
