# Supplementary material for: Association between pain, neuropsychiatric symptoms, and physical function in dementia: a systematic review and meta-analysis
Source: BMC Geriatr. 2015 Apr 19;15:49. doi: 10.1186/s12877-015-0048-6 (PMC4409739; doi:10.1186/s12877-015-0048-6)
Supplement: Additional file 3: — Rating scales for pain that were used in the reported studies. [file 12877_2015_48_MOESM3_ESM.rtf]

Additional file 3. Rating scales for pain that were used in the reported studies

Pain rating scale
	Type of measurement	Reason for development	Studies (first author)
	
Rating of observations
				
PAINAD	Pain Assessment in Advanced Dementia Scale (Warden 2003)	5-item scale (nonverbal behaviours) 
Each item rated on a 3-point scale (0-2)
	Developed to provide a clinically relevant and easy to use pain assessment tool for individuals with advanced dementia
	Leong 2007, Lin 2011 	
DS-DAT	Discomfort Scale-Dementia Alzheimer Type (Hurley 1992)	9-item scale (nonverbal behaviours)
Each item is measured for absence or presence of indicators of discomfort which, if present, are scored for frequency, duration and intensity·	Developed to measure discomfort in elders with dementia of the Alzheimer's type·	Zieber 2005	
Rating of pain based on interview or observation
			
GMPI	Geriatric Multidimensional Pain and Illness Inventory (Clifford 2005)	12-items in 3 subscales: pain and suffering (3), interference (5), emotional distress (4)
Each item rated on a 10-point scale
	Designed to assess pain and its functional, social and emotional consequences, in residents in long term care
	Cipher 2004/2006 	
PPQ	Proxy Pain Questionnaire (Fisher 2002)
	3 items: presence Y/N, frequency, and intensity of pain on 13-point scale
	To assess pain in cognitively impaired older people	Norton 2010 (1 item)	
PGC-PIS
	Philadelphia Geriatric Centre–Pain Intensity Scale 
(Parmelee 1991)	“worst pain” item: highest level of pain experienced over preceding 4 weeks on a 5-point scale 
		Gruber-Baldini 2005, Kunik 2005/Morgan 2012, Williams 2005
	
InterRAI LCTF	InterRAI instrument for Long-Term Care Facilities
	Any type of pain or discomfort in 3 days before assessment, based on extensive evaluation
Items on pain frequency, and severity (0-4)
		Tosato 2011	
MDS	Minimum Data Set 
	Items: pain intensity and frequency
Each item rated on a 3-point scale (0-3)		Ahn 2013, Leonard 2006, Volicer 2009/2011	
Self-report of pain*
				
-
VDS
VRS	Non-verbal visual analogue scale
Verbal Descriptor Scale
Verbal Rating Scale	7-point scale
7-point scale, pain right now 
4-point scale, pain right now 	

	Brummel-Smith 2002 
Shega 2005/2010
Torvik 2010	
* Developed for self-report, but sometimes used in interview
References

	1 	Clifford, P.A. and Cipher, D.J., 2008. The Geriatric Mulitdimensional Pain and Illness Inventory : A New Instrument Assessing Pain and Illness in Long-Term Care. Clinical Gerontologist 28, 45-61.
	2 	Fisher, S.E., Burgio, L.D., Thorn, B.E., Allen-Burge, R., Gerstle, J., Roth, D.L., and Allen, S.J., 2002. Pain assessment and management in cognitively impaired nursing home residents: association of certified nursing assistant pain report, Minimum Data Set pain report, and analgesic medication use. J.Am.Geriatr.Soc. 50, 152-156.
	3 	Hurley, A.C., Volicer, B.J., Hanrahan, P.A., Houde, S., and Volicer, L., 1992. Assessment of discomfort in advanced Alzheimer patients. Res.Nurs.Health 15, 369-377.
	4 	Parmelee, P.A., Katz, I.R., and Lawton, M.P., 1991. The relation of pain to depression among institutionalized aged. J.Gerontol. 46, 15-21.
	5 	Warden, V., Hurley, A.C., and Volicer, L., 2003. Development and psychometric evaluation of the Pain Assessment in Advanced Dementia (PAINAD) scale. J.Am.Med.Dir.Assoc. 4, 9-15.
